# Environmental heterogeneity promotes spatial resilience of phototrophic biofilms in streambeds

**DOI:** 10.1098/rsbl.2018.0432

**Published:** 2018-10-10

**Authors:** Katharine Dzubakova, Hannes Peter, Enrico Bertuzzo, Carmelo Juez, Mário J. Franca, Andrea Rinaldo, Tom J. Battin

**Affiliations:** 1 Stream Biofilm and Ecosystem Research Laboratory, School of Architecture, Civil and Environmental Engineering, Ecole Polytechnique F́edérale de Lausanne, CH-1015 Lausanne, Switzerland; 2 Laboratory of Hydraulic Constructions, School of Architecture, Civil and Environmental Engineering, Ecole Polytechnique F́edérale de Lausanne, CH-1015 Lausanne, Switzerland; 3 Laboratory of Ecohydrology, School of Architecture, Civil and Environmental Engineering, Ecole Polytechnique F́edérale de Lausanne, CH-1015 Lausanne, Switzerland; 4 Department of Environmental Sciences, Informatics and Statistics, Ca’ Foscari University of Venice, 30170 Venice, Italy; 5 River Basin Development Chair Group, Water Science and Engineering Department, IHE Delft Institute for Water Education, 2611 AX Delft, The Netherlands

**Keywords:** biofilm, periphyton, spatial resilience, disturbance, recovery, environmental heterogeneity

## Abstract

The loss of environmental heterogeneity threatens biodiversity and ecosystem functioning. It is therefore important to understand the relationship between environmental heterogeneity and spatial resilience as the capacity of ecological communities embedded in a landscape matrix to reorganize following disturbance. We experimented with phototrophic biofilms colonizing streambed landscapes differing in spatial heterogeneity and exposed to flow-induced disturbance. We show how streambed roughness and related features promote growth-related trait diversity and the recovery of biofilms towards carrying capacity (CC) and spatial resilience. At the scale of streambed landscapes, roughness and exposure to water flow promoted biofilm CC and growth trait diversity. Structural equation modelling identified roughness, post-disturbance biomass and a ‘neighbourhood effect’ to drive biofilm CC. Our findings suggest that the environment selecting for adaptive capacities prior to disturbance (that is, memory effects) and biofilm connectivity into spatial networks (that is, mobile links) contribute to the spatial resilience of biofilms in streambed landscapes. These findings are critical given the key functions biofilms fulfil in streams, now increasingly experiencing shifts in sedimentary and hydrological regimes.

## 1. Introduction

The study of how the environment affects the resilience of ecological systems has rapidly expanded over the last years [1–3]. Most studies on ecological resilience have focused on discrete systems [4,5], with only few considering the spatial resilience of complex landscape matrices [1,2,6,7]. Theory posits that ecological memory (or legacy) and spatial connectivity promote spatial resilience in landscapes [1,2,6,7]. Empirical evidence for spatial resilience remains poorly documented [1,3,6], particularly for microorganisms.

In analogy to plants covering land surfaces, phototrophic biofilms (or periphyton) colonize benthic sediments in streams. They are diverse microbial communities containing algae, prokaryotes and eukaryotes, and they are prime sites of primary production, and carbon and nutrient cycling, even with implications for global biogeochemistry [8]. Understanding how flow-induced disturbance, nutrients, light and grazing control phototrophic biofilms has been an enduring challenge in stream ecology [9,10]. Stream biofilms are recognized as microbial landscapes [11] that colonize streambed landscapes steadily reconfigured by the interaction between sediments and water flow [12]. The iteration of a structure–function coupling across such sedimentary and microbial scales was hypothesized to contribute to the fitness of biofilms in streams [8]. However, poor understanding of the coupling between sedimentary structures and biofilms [9,13,14] precludes predictions of the impacts of shifting sedimentary [15] and hydrological [16] regimes on the resilience of microbial life in streams.

In this letter, we test the hypothesis that the three-dimensional heterogeneity of streambeds and related hydraulics affect the spatial resilience of phototrophic biofilms as regards their capability to reorganize and achieve carrying capacity (CC) following flow-induced disturbance. CC encapsulates environmental constraints with growth dynamics and biogeochemical fluxes and is therefore a key quantity in ecology.

## 2. Material and methods

### (a) Experimental design and data acquisition

We grew phototrophic biofilms in an outdoor flume containing 10 streambed landscapes (0.2 × 0.4 m) with different topographies and near-bottom fluid dynamics configured from sediments differing in size (electronic supplementary material, S1). After 13 days of biofilm growth at constant flow, we simulated a storm event disturbing and partially eroding biofilms in all landscapes. We monitored post-disturbance biofilm recovery over 28 days using time-lapsed imaging with a modified digital camera. Using the different optical bands, we calculated the normalized difference vegetation index (NDVI) as a proxy for phototrophic biomass without resolving for biodiversity [17] (electronic supplementary material, S1). We quantified community-aggregated growth traits of the biofilms by fitting a logistic growth curve to pixel-level (0.2 mm) NDVI data (electronic supplementary material, S1). From more than 4 × 10^6^ growth curves, we extracted lag phase, as the time required for the post-disturbance reorganization of the biofilms, recovery through growth, and CC as the transient equilibrium reached through recovery. Lag phase, growth and CC are important fitness components of microorganisms in fluctuating environments [18] and traits relevant to ecosystem functioning [19]. We related these traits to geomorphic characteristics of the streambed as derived from a digital elevation model (DEM) and to flow velocity and near-bottom shear stress derived from hydraulic modelling. Concave and convex microhabitats within the streambed landscapes were identified using a discrete Laplacian operator (electronic supplementary material, S1) [20].

### (b) Statistical analyses

We computed response diversity of growth traits as the multivariate inhomogeneous intensities (*Φ*) [21,22] of lag phase, recovery rate and CC at pixel scale in all landscapes (electronic supplementary material, S1); low *Φ* values indicate high diversity of growth traits. Spatially explicit multiple linear regressions were computed to estimate the contributions of topography, hydraulics and post-disturbance biomass to growth-related traits in each landscape. Models included a ‘neighbourhood effect’ reflecting a positive effect of maxima in post-disturbance biomass close to the focal pixel and thus the effects of near-distance dispersal on spatial recovery. The models further included a spatially explicit measure of the topographic embeddedness within the streambed landscape. Model results scaffolded a piecewise structural equation model (SEM) [23,24]. SEM served to resolve the networks of relationships among environmental variables and growth traits possibly driving biofilm CC and resilience in the streambed landscapes.

## 3. Results and discussion

Streambed topographic roughness explained 51% (*p* = 0.01) of the variation in average biofilm CC across all landscapes (electronic supplementary material, S2 and S3). Average biofilm CC was consistently higher in concave than in convex microhabitats (paired *t*-test, mean of differences: 0.039, *t* = 17.6, d.f. = 9, *p* < 0.001), which may be attributable to lower shear stress and hence elevated protection from flow-induced erosion in concave microhabitats. These findings highlight topographic roughness as a control on biofilm CC at the level of streambed landscapes.

**Figure 1.**
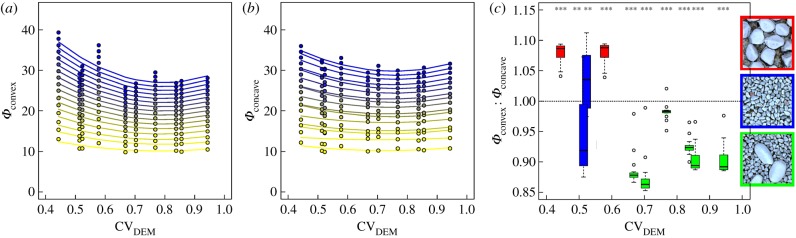
Diversity of growth traits of phototrophic biofilms in streambed landscapes. (*a*,*b*) Relationships between inhomogeneity intensity (*Φ*) based on lag phase, recovery rate and carrying capacity, and topographic roughness (CV_DEM_) for increasingly decomposed trait spaces (number of bins ranging from 5^3^, yellow, to 20^3^, blue, corresponding to 2480 and 39 data per bin, respectively); data were fitted using second-order polynomes. (*c*) *Φ*_convex_ : *Φ*_concave_ values less than 1 indicate lower trait diversity in concave than in convex microhabitats; asterisks (**p < 0.01, ***p < 0.001) indicate significant differences from 1 (Wilcoxon test).

To further test the effect of roughness features on the response diversity of community-aggregated growth traits, we compared *Φ* for concave (*Φ*_concave_) and convex (*Φ*_convex_) microhabitats separately (electronic supplementary material, S4). We found a nonlinear relationship between trait diversity and topographic roughness independent of microhabitat type (figure 1*a,b*). This pattern was particularly pronounced for smaller bin sizes of the trait space (greater than 15). Trait diversity was lowest in landscapes with reduced topographic roughness, but increased and levelled off with increasing streambed roughness. As indicated by the ratios (less than 1) of *Φ*_convex_ : *Φ*_concave_ (figure 1*c*), growth trait diversity was generally lower in concave than in convex microhabitats in streambeds with elevated topographic roughness (i.e. CV_DEM_ > 0.6). Trait diversity was elevated in concave microhabitats in streambeds composed of small or large sediment grains only and thus with reduced roughness (CV_DEM_ < 0.6). These results suggest that local stabilities due to protection in concave microhabitats select for biofilm assemblages with less diverse growth traits and consequently reduced response diversity. Reduced nutrient replenishment due to boundary layer phenomena in concave microhabitats may select phototrophic biofilms with a slow-growing lifestyle. In contrast, convex microhabitats protruding into the turbulent flow offer opportunities for biofilms with more diverse growth traits. These findings point at the relevance of environmental heterogeneity for the diversification of biofilm growth traits and their potential for spatial resilience—a relationship that has been postulated by theory [7,25] but not yet shown for microbial systems.

**Figure 2.**
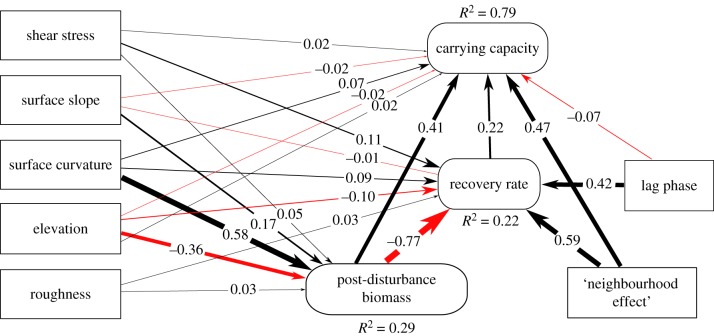
SEM representing connections between physical and biological parameters contributing to phototrophic biofilm resilience in streambed landscapes. Black arrows represent positive effects, and red arrows represent negative effects. The numbers denote the relative effect sizes scaling with the arrow thickness. The goodness of fit of the SEM was evaluated based on Fisher *c* = 144.59, d.f. = 4, *p* < 0.001 and Akaike information criterion AIC = 200.6.

SEM (electronic supplementary material, S5 and S6) demonstrated the relative importance of local memory effects and mobile links among neighbouring patches for biofilm spatial resilience (figure 2). Flow and the exposure to shear stress depending on surface curvature may generate biological legacies of biofilms owing to biomass accumulation in protected microhabitats. This notion is supported by the positive relationship between post-disturbance biomass and surface curvature (*β* = 0.58) as well as elevation and hence the exposure to shear stress (*β* = −0.36). SEM results further suggest that topographic embeddedness enhances post-disturbance biomass, which in turn enhances recovery towards CC (*β* = 0.41). These findings support the patterns observed at landscape scale on biofilms protected from flow-induced disturbance in concave microhabitats and implies that less affected biofilms function as sources for the re-colonization of adjacent patches. Such an ecological memory effect [1,2,6] promotes the capability of biofilms to reach CC at the scale of the entire streambed landscape. This notion is supported by a ‘neighbourhood effect’ serving as a proxy for near-distance dispersal, which is strongly related to CC (*β* = 0.47) and recovery rate (*β* = 0.59). This is analogous to a rescue effect [26] or mobile links [1,2,6] where dispersal contributes to the spatial resilience of biofilms at the scale of streambed landscapes.

The negative relationship (*β* = −0.77) between post-disturbance biomass and recovery suggests trade-offs between protection from shear stress, particularly in concave microhabitats, and reduced nutrient replenishment therein [27]. In addition, the coupling (*β* = 0.42) between lag phase and recovery indicates rapid growth after an extended lag phase, translating into a slow-but-efficient growth strategy of phototrophic biofilms during recovery. A similar pattern was described for stream biofilm-forming microorganisms, where initially slow growth likely occurred at the cost of the production of the biofilm matrix [28]. At the same time, the weak relationship (*β* = 0.22) between recovery and CC suggests dispersal (as the ‘neighbourhood effect’) and ecological memory effects, rather than active growth, to be important for the spatial resilience of biofilms.

In summary, our findings unravel phototrophic biofilms as networks of local patches that are spatially connected in the three-dimensional matrices of streambed landscapes. This configuration may modulate the spatial resilience of biofilms, thereby contributing to their stability in an environment that is becoming increasingly exposed to shifts in the sedimentary and hydrological regimes.

## Supplementary Material

Supplementary material

## Data accessibility

Data are available via Figshare (https://doi.org/10.6084/m9.figshare.c.4226819.v1).
