# Supplementary material for: Environmental heterogeneity promotes spatial resilience of phototrophic biofilms in streambeds
Source: Biol Lett. 2018 Oct 10;14(10):20180432. doi: 10.1098/rsbl.2018.0432 (PMC6227859; doi:10.1098/rsbl.2018.0432)
Supplement: Supplementary material [file rsbl20180432supp1.pdf]

Supplementary Material for

Environmental heterogeneity promotes spatial resilience of phototrophic biofilms in streambeds

Katharine Dzubakova, Hannes Peter, Enrico Bertuzzo, Carmelo J. Juez, Mario Franca, Andrea Rinaldo, Tom J. Battin

Correspondence to: tom.battin@epfl.ch

<ins>Overview of Supplementary Material:</ins>

Supplementary Material S1 provides the full Materials and Methods

Supplementary Material S2 shows relationships between landscape DEM CV and carrying capacity as well as the distribution of shear stress among landscape components.

Supplementary Material S3 provides visual representations of measured and modelled parameters for each landscape.

Supplementary Material S4 contains a visual explanation of the conceptual basis of response trait diversity as measured from multivariate inhomogeneous intensities and response trait diversity in concave and convex landscape components.

Supplementary Material S5 includes regression statistics for multiple linear regressions.

Supplementary Material S6 provides model parameter and evaluation of the piece-wise structural equation model.

Supplementary Material S7 provides test statistics comparing median $\Phi_{convex}:\Phi_{concave}$ against a value of 1.

Supplementary Material S1

Material and methods

Experimental design. Ten streambed landscapes (0.2 × 0.4 m) were constructed in a recirculating outdoor flume. We used different compositions of sediment of two size categories (diameters ranging from 15-25 mm and from 60-100 mm) to achieve a gradient of topographic heterogeneity, expressed as the coefficient of variation (CV) of the digital elevation model (DEM). Values of CV_{DEM} ranged from 0.44 to 0.58 for landscapes with only small and large grains, respectively, to 0.86 to 0.96 for landscapes with mixed grain sizes. We grew benthic phototrophic biofilms from natural surface water and exposed to natural light; major algae contained in the biofilms were diatoms and green algae. Discharge was set at 8 L s^{-1} yielding an average flow velocity of 0.7 m s^{-1}; honey-comb systems ensured reproducible flow over each of the landscapes. Exposed to the same inoculum and water chemistry we let biofilms to establish over 13 days before we induced a physical disturbance mimicking a storm event and partially eroding phototrophic biofilms. Similar flume settings[1] and experimentally induced disturbance[2] were previously used to study biofilm ecology.

Biomass recovery. The recovery of biofilms in all ten streambed landscapes was monitored on a daily basis for 18 days until majority of biofilms reached maturity and then on the day 28. To achieve high-resolution spatial coverage of the biomass distribution we determined the dimensionless Normalized Difference Vegetation Index (NDVI) as widely used for terrestrial vegetation cover without resolving for biodiversity[3]. Imaging was done with a modified digital camera (Canon 7D mark II, hot mirror filter removed) mounted on a slider. The camera was equipped with a 22.4 × 15.0 mm sensor, a Canon EF 24 mm f/2.8 IS USM Lens with three filters: a colour correction filter (XNiteCC158: centre wavelength: 483 nm; 50% transmission: 325 nm, 645 nm), and two bandpass filters (XNiteBPB58: 660 nm-750 nm, XNiteBPG58: 710 nm-855 nm). For image acquisition, landscapes were shaded from natural light and illuminated by two halogen lamps (100 W, Osram) to obtain comparable light conditions in space and time. Data were stored in CR2 format and converted to RAW TIFF (16bit) without gamma correction, demosaicing or scaling (DCRAW software). Multimodal affine image registration with 500 iterations was used to align images. To obtain robust data, images (1 pixel = 0.1 mm) were resized by a factor of 0.5 resulting in a precision of 1 pixel (corresponding to 0.2 mm).

NDVI for each pixel and point in time was calculated as

$$\mathrm{NDVI} = \frac{(NIR_{660-750nm} + NIR_{710-855nm} - green_{425-625\mathbf{nm}})}{(NIR_{660-750nm} + NIR_{710-855nm} + green_{425-625\mathbf{nm}})},$$

where *NIR* is the near-infrared light and $green_{425\text{-}625\ \mathrm{nm}}$ refers to the green light range defined by the camera sensitivity. After the selection of images without shade, we used a five-day moving median filter and a time-series outlier detection algorithm (*tsoutlier* implemented in R) prior to statistical analyses of the NDVI data.

Post-disturbance biomass was estimated as the median NDVI of the first three days after perturbation. Phototrophic biomass recovery towards CC was modelled from normalized NDVI data by fitting logistic curves (R package *grofit*):

$$NDVI(t) = NDVI(t=0) + \frac{A}{1+\exp\left(\frac{4r}{A}(\lambda - t) + 2\right)},$$

where t [day] represent time after disturbance, r [day^{-1}] the maximum biomass growth rate (i.e. recovery rate) , λ [day] the lag phase and $A=CC\text{-}NDVI(t=0)$. Bootstrap and cross-validation techniques were used for estimating confidence intervals providing means and standard deviation for each fitted parameter. The model converged, indicating that the growth response was well described by a logistic curve for 21% to 33% of all pixels in each landscape accounting for 6.5×10^6 growth curves in total. We did not expect to obtain the fit for the entire streambed landscape because of bare sediment surfaces. We excluded pixels with a standard deviation of recovery rate > 0.004 [day^{-1}] as computed from bootstrapping. As result, we retained more than 4×10^6 growth curves from which we used lag phase, recovery and CC as relevant growth traits.

Digital elevation model. Structure for motion photogrammetry was used to construct a DEM for each streambed landscape. For each landscape, 25 images were recoded from a hemisphere perspective from which DEMs were constructed using PhotoScan (Agisoft), scaled and rectified in MeshLab[4], linearly transformed (Matlab 2015b) and referenced in ArcGIS (10.3). The final DEM had a resolution of 1 mm and an average precision < 2 mm. Convex and concave landscape components were identified using a discrete Laplacian operator using a 5-point-stencil on surface elevation using a threshold of 10^{-6}.

Fluid dynamics. A 2-dimensional depth-averaged numerical scheme[5] developed for unsteady flows was used to simulate the flow, particularly local velocities and surface shear stress, for each streambed landscape. Depth-averaged mass and momentum equations were solved by a numerical scheme using a finite volume method based on a Roe-type solver. The surface shear stress corresponds to the momentum exchanges between the free-slip upper flow and the rough lower boundary and closes the momentum balance in one finite volume of flow discretization. Hydrostatic pressure distributions are considered in the moment balance equations, which is admissible given the smooth curvature of the streamlines in the flow. The model was calibrated and validated with experimental results from flow velocities determined with 3D Acoustic Doppler Velocimetry (Nortek) and water surface elevations.

Trait response diversity. We calculated the inhomogeneous intensity as the multivariate variability (var_m) of lag phase (λ), recovery rate (r) and CC (K) for pixels in all landscapes as $var_m = (\iiint(\rho_r(\lambda,r,K) - \rho_a)^2\, d\lambda\, dr\, dK)^{1/2}$ in the domain D defined by lim(var), where $\rho_r(\lambda, r, K)$ is density of trait triplets around (λ, r, K) and ρ_a is the average density across the trait space[6]. The multivariate variability, var_m, was numerically approximated by splitting the trait space into uniformly spaced bins. Sensitivity to differences in trait distribution increases with decreasing bin size. We estimated var_m using 5 to 20

subintervals along each of the three trait space dimensions, reflecting 125 to 8000 bins. We used a maximum of 8000 divisions because the average pixel count per bin dropped below 50.

Spatially explicit parameters. We computed one biological and one physical spatially explicit parameter to be used in the multiple linear regression analyses (see below). First, the "neighbourhood effect" is conceptualized to reflect a positive association between adjacent biomass pixels through near-distance dispersal. Assuming biomass isotropy, we set a maximum distance of a "neighbourhood effect" around each pixel based on local Moran's I statistics[7]. For this, the maximal similarity distance of post-disturbance biomass was identified from spatial correlograms computed for 25 equally distributed distances from 1 to 25 mm. The "neighbourhood effect" was estimated as a distance-weighted Gaussian function of post-disturbance biomass; the kernel size was set to the maximal similarity distance and the standard deviation of the Gaussian distribution was 3 pixels. Conceptually, this effect is greatest if large post-disturbance biomass is close to the focal pixel. Second, we computed topographic roughness for each pixel to contextualize its topographic embeddedness within the streambed landscape. We defined roughness as the sum of coefficients of variation of surface elevation for increasing surrounding area (diameter from 5 to 100 mm by increment of 5 mm). We used a kernel size up to 100 mm to approximate large-scale impacts of topographic roughness.

Spatially explicit multiple linear regression analyses. Spatially explicit multiple linear regression analysis was computed to estimate the contributions of topography, hydraulics and post-disturbance biomass parameters on MCC and related growth-related traits for each landscape. Results formed the basis for the structural equation model (see below). Both the "neighbourhood effect" and topographic embeddedness made the models spatially explicit. The local Moran's I avoids problems of non-stationary autocorrelation structure and spatial trend in the data. Unlike spatial models[8,9], we did therefore not compute spatial autocorrelation of biomass from regression error or from CC as response variable, but from post-disturbance biomass as an explanatory variable. We assumed that spatial autocorrelation of post-disturbance biomass directly translated into final biomass structure. The relative contribution of individual variables to explain variance in linear models was quantified using LMG metrics as implemented in the *R* package *relaimpo*[10]. This metrics decomposes R^2 into non-negative contributions that automatically sum to the total R^2. The approach is based on sequential coefficients of determination (R^2) and takes into account the dependence on orderings by averaging over orderings using simple unweighted averages[10]. Variance inflation factors and Belsley collinearity diagnostics were used to identify collinear terms. Surface shear stress and flow velocity were significantly collinear (VIF < 10) and we therefore included only surface shear stress in the regression analyses.

Structural equation model. A piecewise structural equation model (SEM) was computed using *piecewiseSEM*[11] to infer relationships between topographic, hydraulic and biological drivers of biofilm resilience. We used simple multivariate linear models in accordance to our multiple linear

regression analyses to scaffold the SEM structure. Input data to the regressions were neither scaled nor transformed. Goodness-of-fit statistics for linear models was evaluated by R^2 for component models in SEM. SEM enabled us to identify missing paths, which were subsequently implemented in the redesigned list of regressions representing the SEM. We then re-computed the SEM. Independence claims for SEM (goodness-of-fit test for piecewise SEM) were evaluated by Fisher's C statistics, Chi-squared test degrees of freedom and the outcome (p-value) of the significance test derived from a Chi-squared distribution. Moreover, the Akaike Information Criterion score (AIC) and the likelihood degrees of freedom (K) were computed. To retrieve causal path coefficients from SEM, variables were scaled by mean and variance, and the factor variables were left in their original units. The path coefficients were described by causal path (response, predictor), coefficient estimate, standard error, and associated p-value. Significance tests (i.e., P-values) for correlated errors were derived from a t-distribution[12].

References

1. Singer, G., Besemer, K., Schmitt-Kopplin, P., Hödl, I. & Battin, T. J. 2010 Physical Heterogeneity Increases Biofilm Resource Use and Its Molecular Diversity in Stream Mesocosms. *PLoS ONE* **5**, e9988–11. (doi:10.1371/journal.pone.0009988)
2. Cardinale, B. J. 2011 Biodiversity improves water quality through niche partitioning. *Nature* **472**, 86–89. (doi:10.1038/nature09904)
3. Pettorelli, N., Vik, J. O., Mysterud, A., Gaillard, J.-M., Tucker, C. J. & Stenseth, N. C. 2005 Using the satellite-derived NDVI to assess ecological responses to environmental change. *Trends in Ecology & Evolution* **20**, 503–510. (doi:10.1016/j.tree.2005.05.011)
4. Cignoni, P., Callieri, M. & Corsini, M. 2008 Meshlab: an open-source mesh processing tool. *Eurographics Italian …*
5. Juez, C., Murillo, J. & García-Navarro, P. 2014 A 2D weakly-coupled and efficient numerical model for transient shallow flow and movable bed. *Advances in Water Resources* **71**, 93–109. (doi:10.1016/j.advwatres.2014.05.014)
6. Baddeley, A. 2009 Analysing spatial point patterns in\'R\'. (doi:10.1234/12345678)
7. Anselin, L. 1995 Local indicators of spatial association—LISA. *Geographical Analysis* (doi:10.1111/j.1538-4632.1995.tb00338.x)
8. Beale, C. M., Lennon, J. J., Yearsley, J. M., Brewer, M. J. & Elston, D. A. 2010 Regression analysis of spatial data. *Ecology Letters* **13**, 246–264. (doi:10.1111/j.1461-0248.2009.01422.x)
9. Dormann, C. F. et al. 2007 Methods to account for spatial autocorrelation in the analysis of species distributional data: a review. *Ecography* **30**, 609–628. (doi:10.1111/j.2007.0906-7590.05171.x)
10. Grömping, U. 2006 Relative Importance for Linear Regression inR: The Packagerelaimpo. *Journal of Statistical Software* **17**. (doi:10.18637/jss.v017.i01)
11. Lefcheck, J. S. 2015 piecewiseSEM: Piecewise structural equation modelling inr for ecology, evolution, and systematics. *Methods in Ecology and Evolution* **7**, 573–579. (doi:10.1111/2041-210x.12512)
12. Shipley, B. 2016 *Cause and Correlation in Biology*. (doi:10.1017/cbo9781139979573)

Supplementary Material S2 Relationships between mean carrying capacity and CV_{DEM} for the total landscape (R^2 = 0.57, p = 0.01), convex (R^2 = 0.36, $p < 0.05$) and concave (R^2 = 0.44, $p < 0.05$) microhabitats (*a*); shown are linear regression models (solid lines) and their 95% confidence intervals (dashed lines). Boxplots showing the differences in surface shear stress between concave and convex microhabitats for each streambed landscape (*b*); differences were statistically different across all landscapes. Test statistics of Wilcoxon rank sum tests with continuity correction for differences in median shear stress between convex and concave landscape components for each of the experimental landscapes are given below.

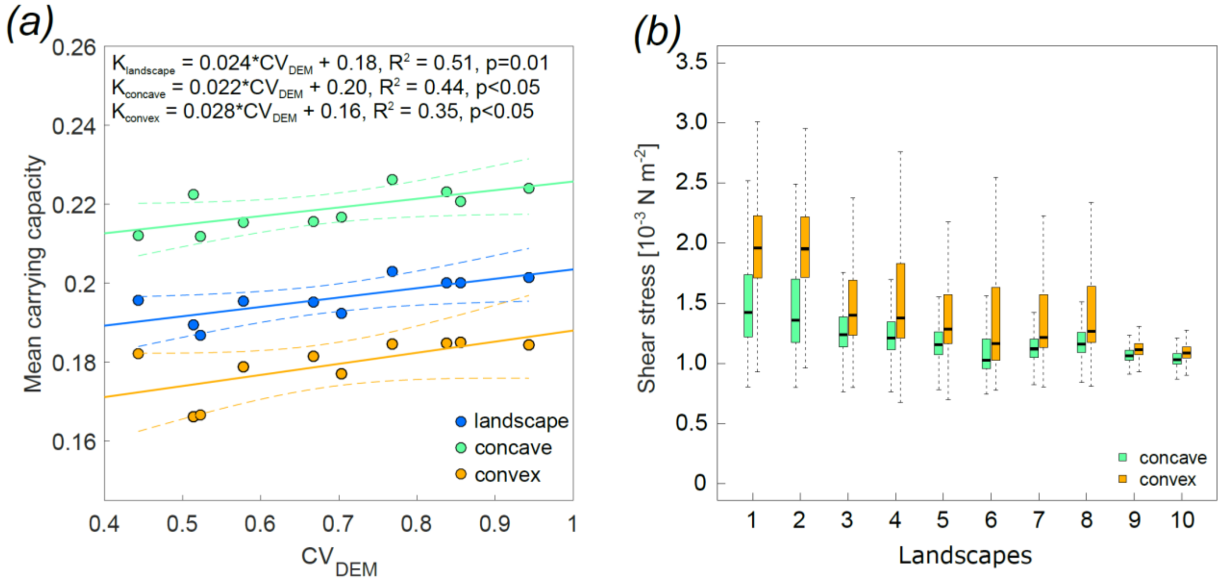

					landscape					
	1	2	3	4	5	6	7	8	9	10
difference in median shear stress (× 10^{-4})	5.36	5.90	1.60	1.64	1.28	1.41	0.94	1.07	0.48	0.55
W (× 10^{11})	2.35	2.05	3.80	3.73	3.64	3.82 [1]	3.70	3.62	3.90	3.76
p value	< 0.01	< 0.01	< 0.01	< 0.01	< 0.01	< 0.01	< 0.01	< 0.01	< 0.01	< 0.01

Supplementary Material S3 Visual representation of measured or modelled parameter for each landscape. Based on the DEM, we estimated elevation (*a*), aspect (*b*), slope (*c*), surface curvature (concave/convex) (*d*) and roughness (*e*). Using hydraulic modelling, we estimated shear stress (*f*) and flow velocity (*g*). Biological parameters include post-disturbance biomass (*h*), lag phase (*j*), recovery rate (*k*), carrying capacity (*l*) and "neighbourhood effect" (*m*).

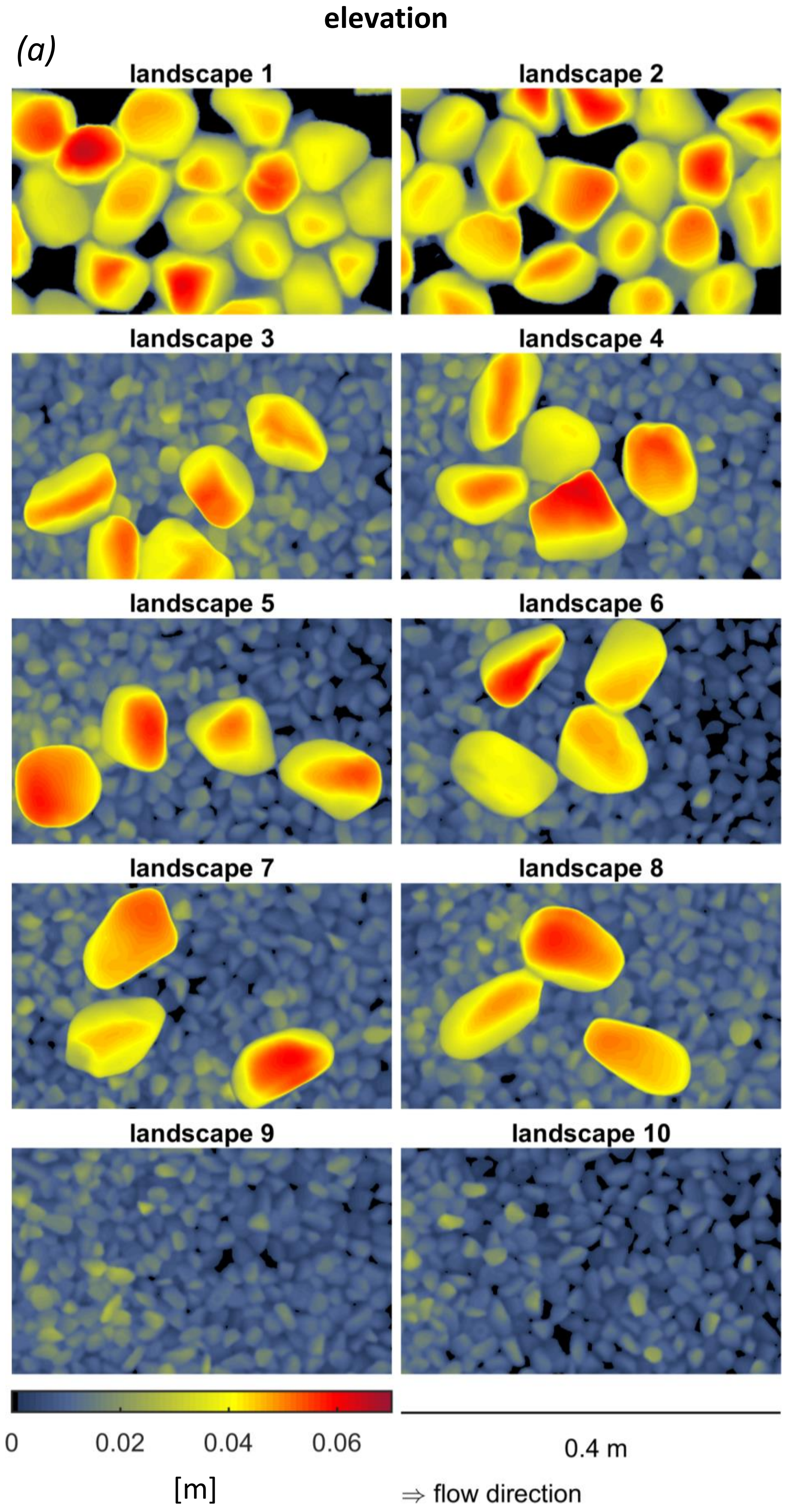
elevation
(a)
landscape 1
landscape 2
landscape 3
landscape 4
landscape 5
landscape 6
landscape 7
landscape 8
landscape 9
landscape 10
0
0.02
0.04
0.06
[m]
0.4 m
⇒ flow direction

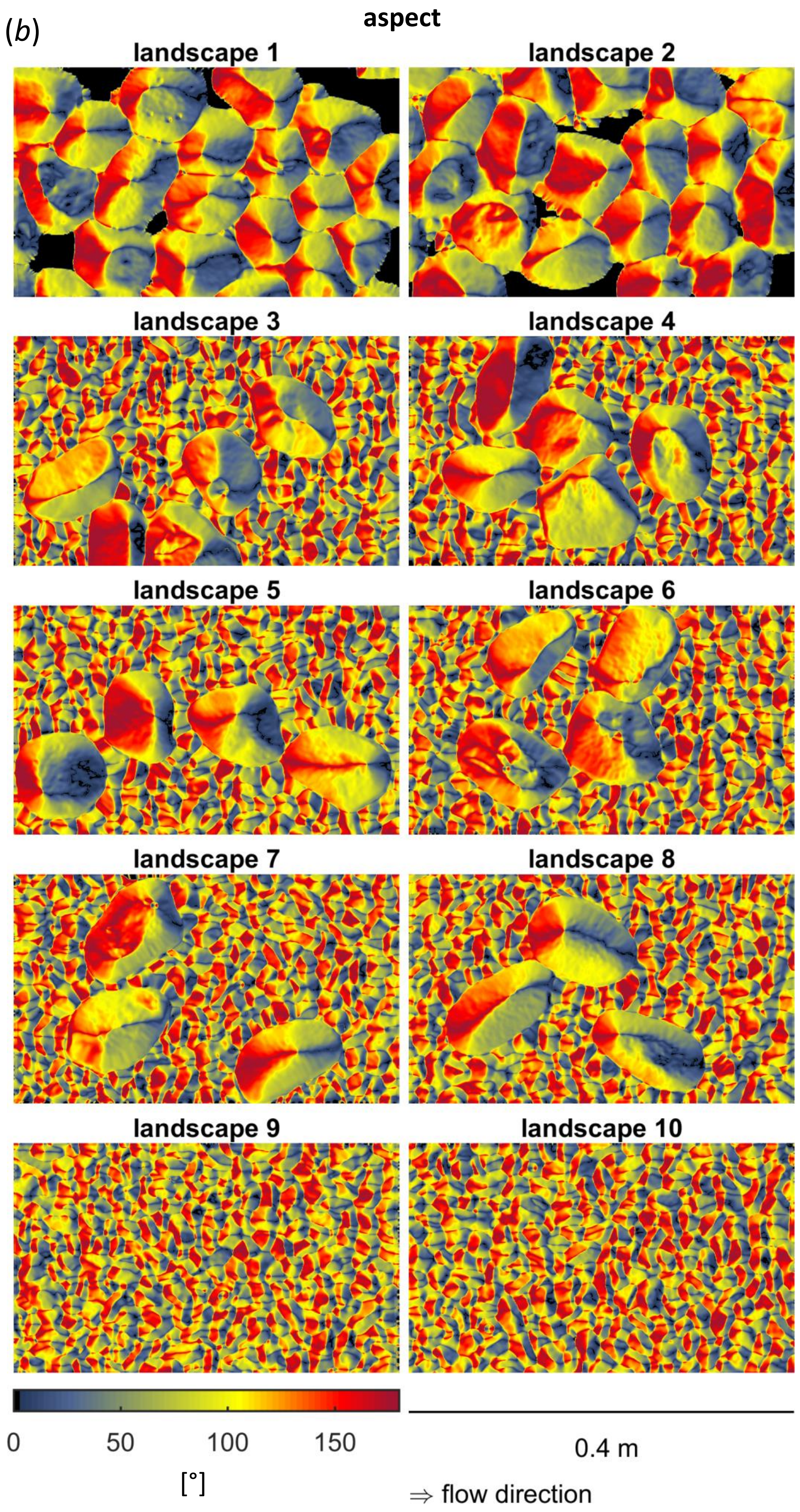
(b)
aspect
landscape 1
landscape 2
landscape 3
landscape 4
landscape 5
landscape 6
landscape 7
landscape 8
landscape 9
landscape 10
0
50
100
150
[°]
0.4 m
⇒ flow direction

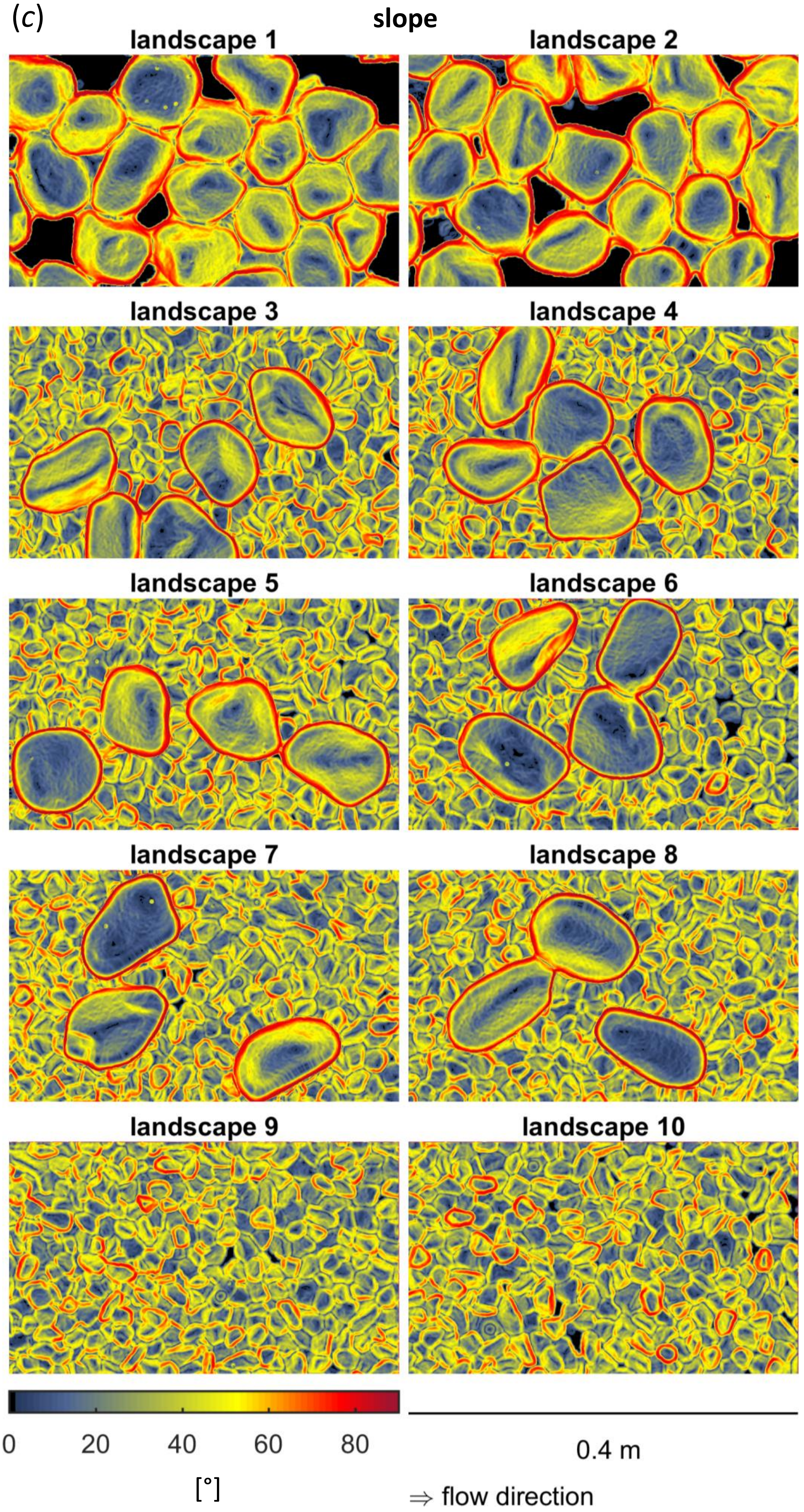
(c)
slope
landscape 1
landscape 2
landscape 3
landscape 4
landscape 5
landscape 6
landscape 7
landscape 8
landscape 9
landscape 10
0
20
40
60
80
[°]
0.4 m
⇒ flow direction

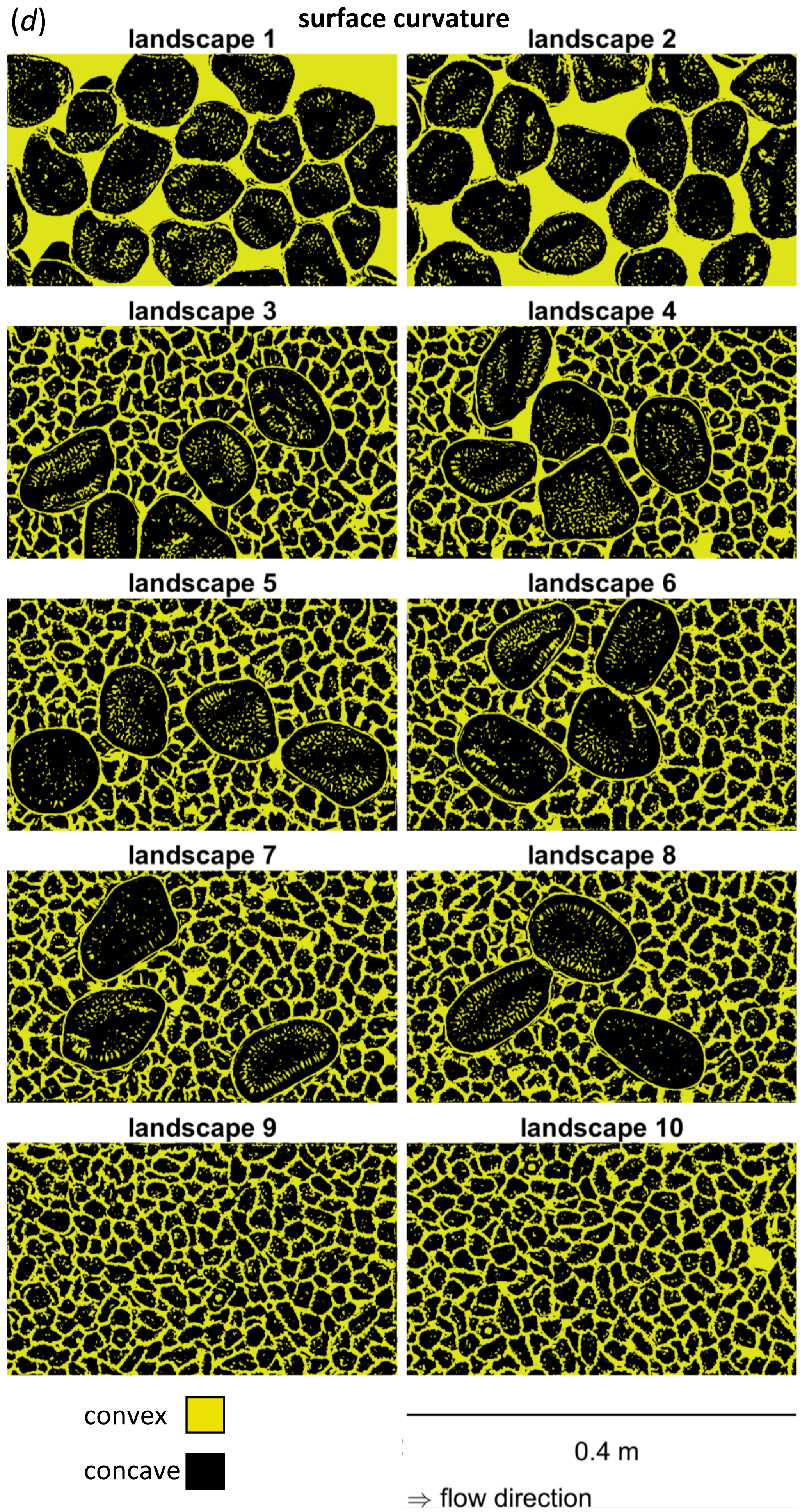
(d)
surface curvature
landscape 1
landscape 2
landscape 3
landscape 4
landscape 5
landscape 6
landscape 7
landscape 8
landscape 9
landscape 10
convex
concave
0.4 m
⇒ flow direction

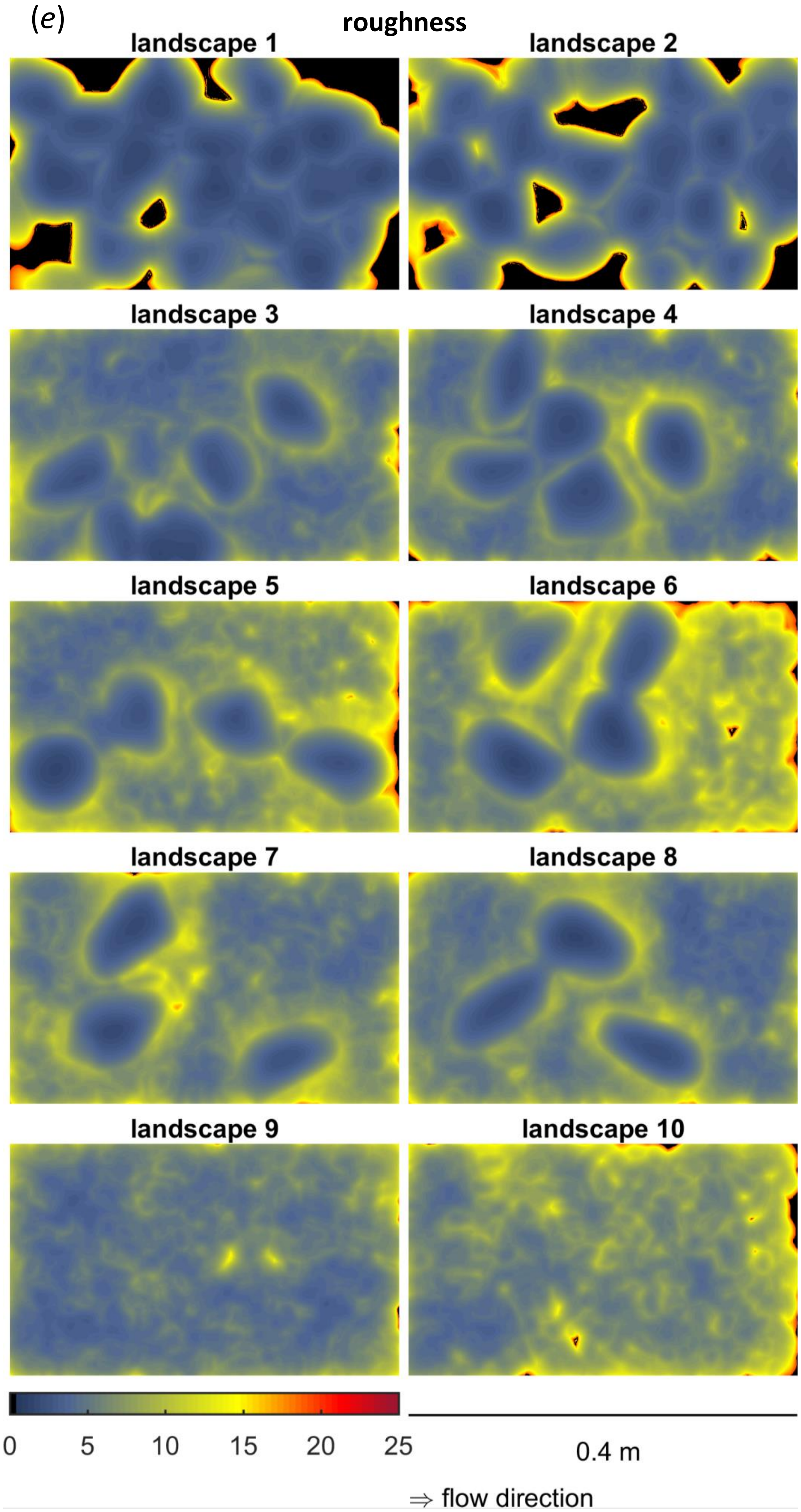

(e)
roughness
landscape 1
landscape 2
landscape 3
landscape 4
landscape 5
landscape 6
landscape 7
landscape 8
landscape 9
landscape 10
0
5
10
15
20
25
0.4 m
⇒ flow direction

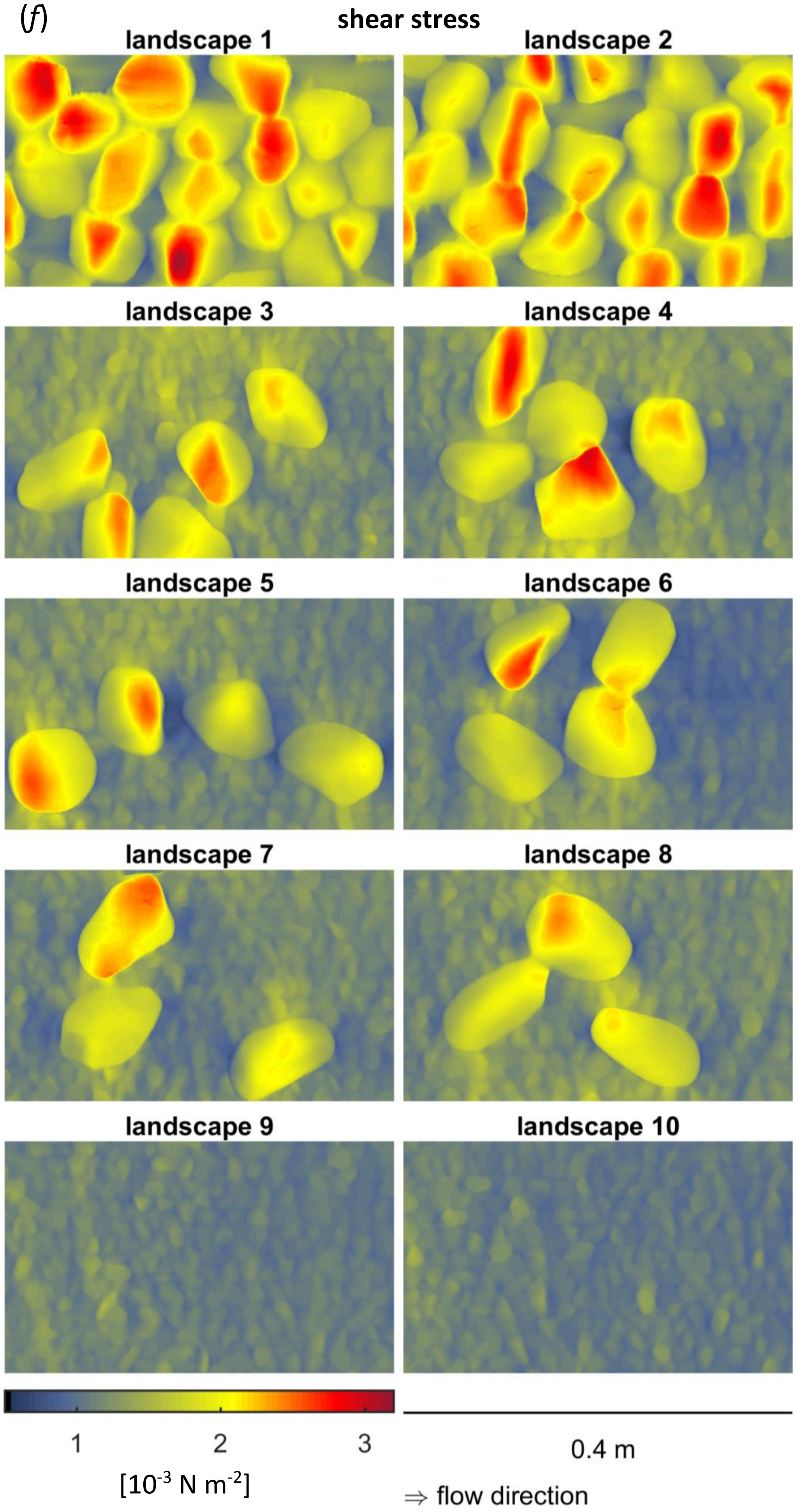
(f)
shear stress
landscape 1
landscape 2
landscape 3
landscape 4
landscape 5
landscape 6
landscape 7
landscape 8
landscape 9
landscape 10
1
2
3
[10-3 N m-2]
0.4 m
⇒ flow direction

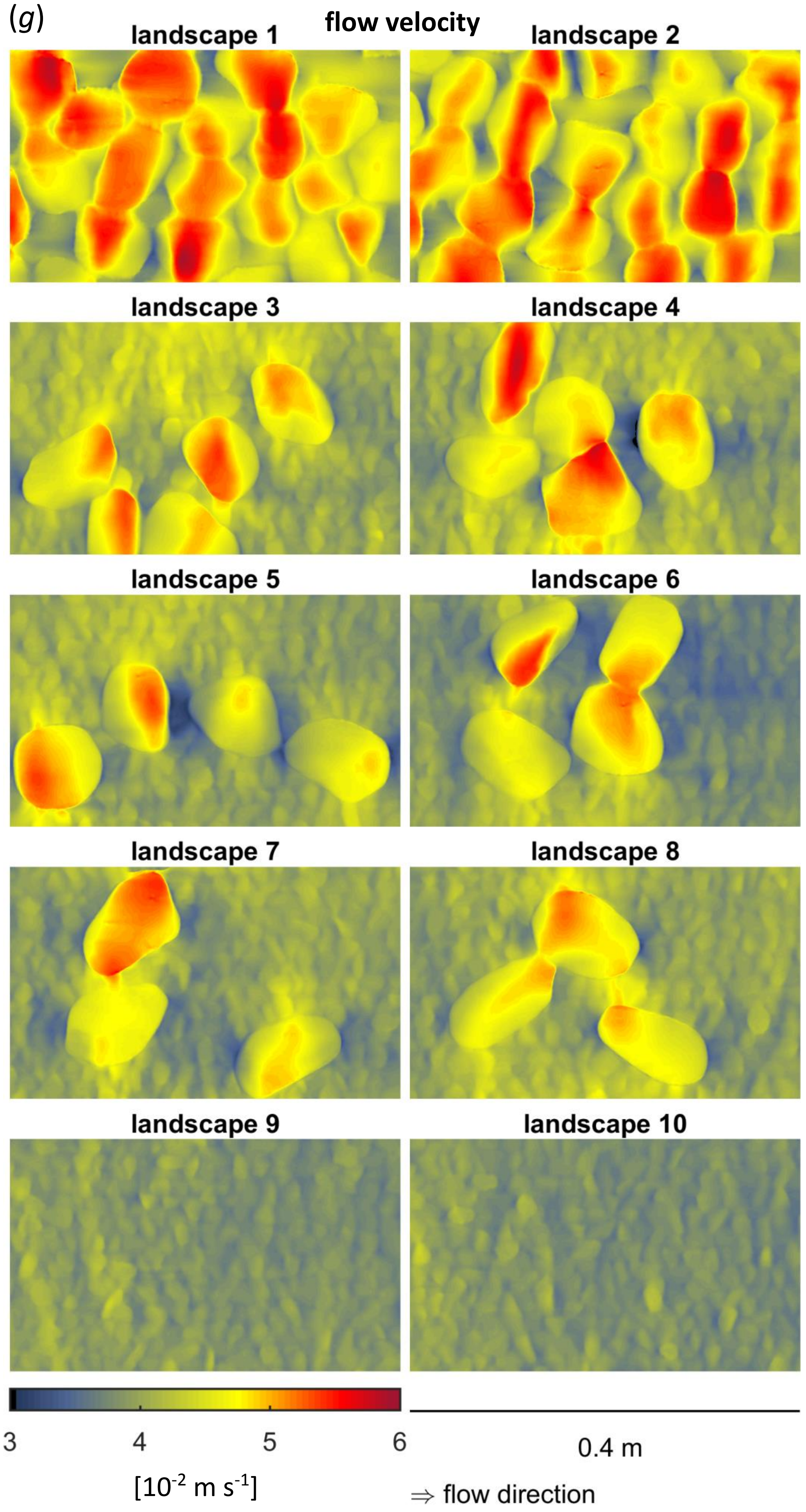
(g)
flow velocity
landscape 1
landscape 2
landscape 3
landscape 4
landscape 5
landscape 6
landscape 7
landscape 8
landscape 9
landscape 10
3
4
5
6
[10-2 m s-1]
0.4 m
⇒ flow direction

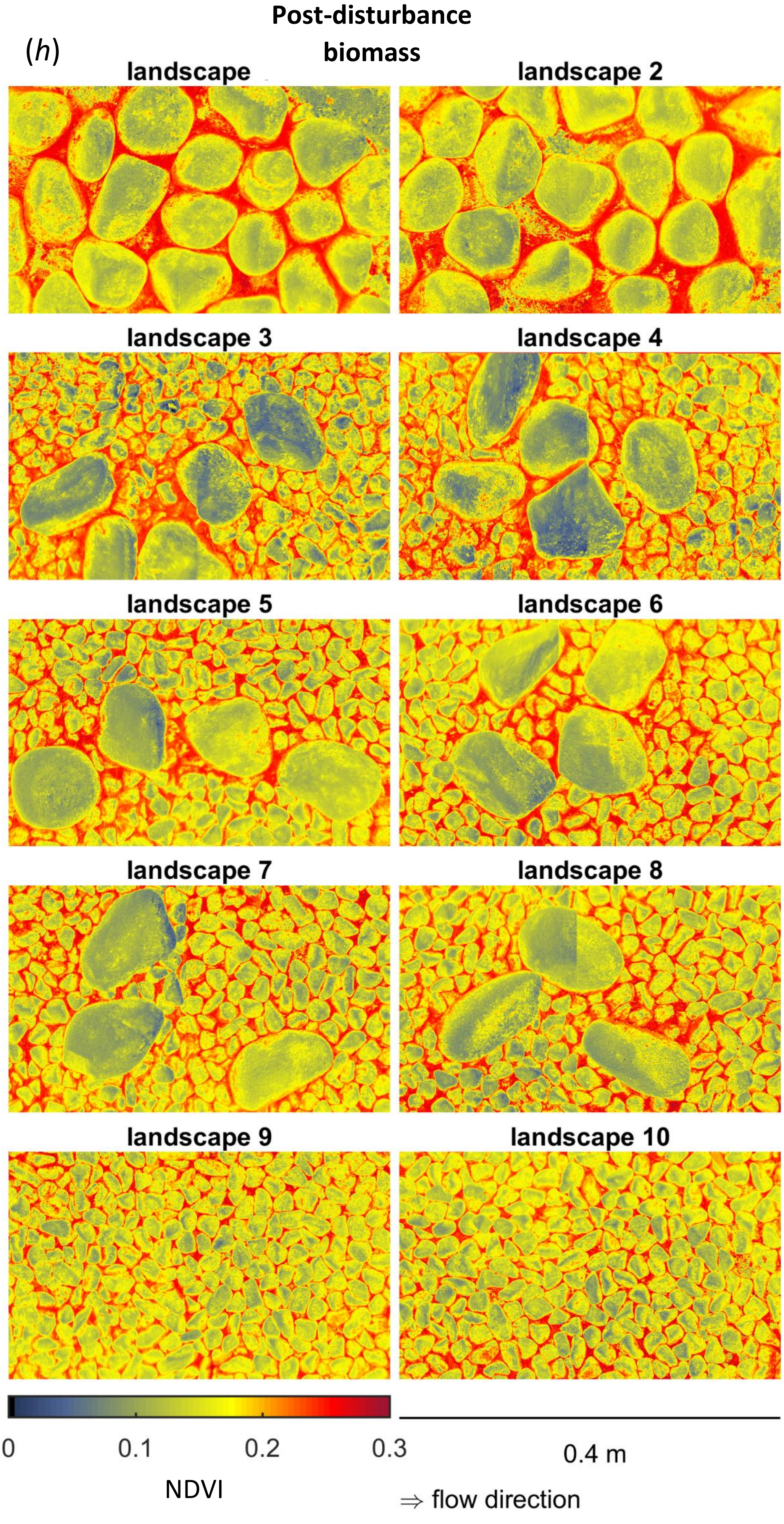
Post-disturbance
biomass
(h)
landscape
landscape 2
landscape 3
landscape 4
landscape 5
landscape 6
landscape 7
landscape 8
landscape 9
landscape 10
0
0.1
0.2
0.3
NDVI
0.4 m
⇒ flow direction

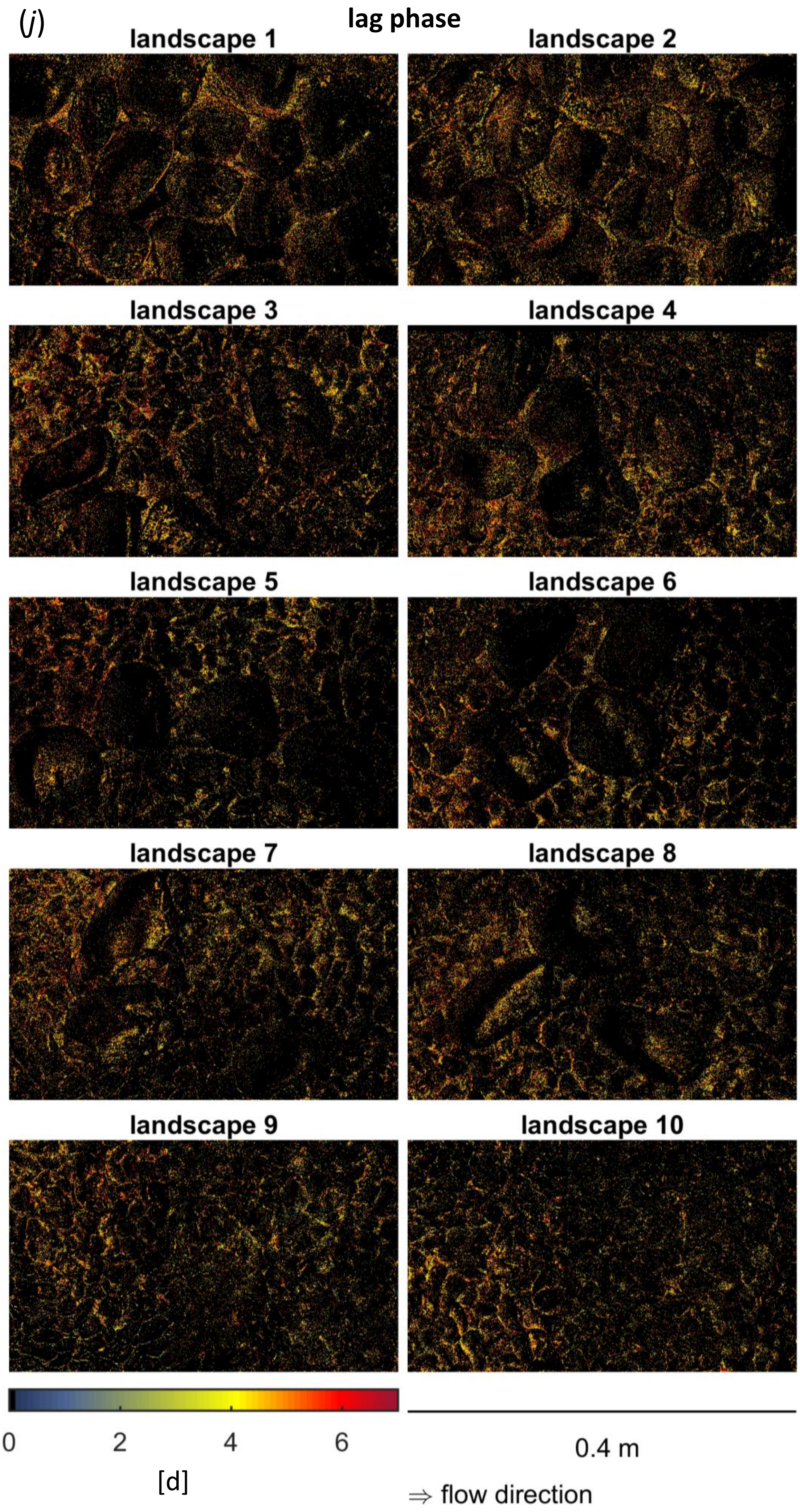
(j)
lag phase
landscape 1
landscape 2
landscape 3
landscape 4
landscape 5
landscape 6
landscape 7
landscape 8
landscape 9
landscape 10
0
2
4
6
[d]
0.4 m
⇒ flow direction

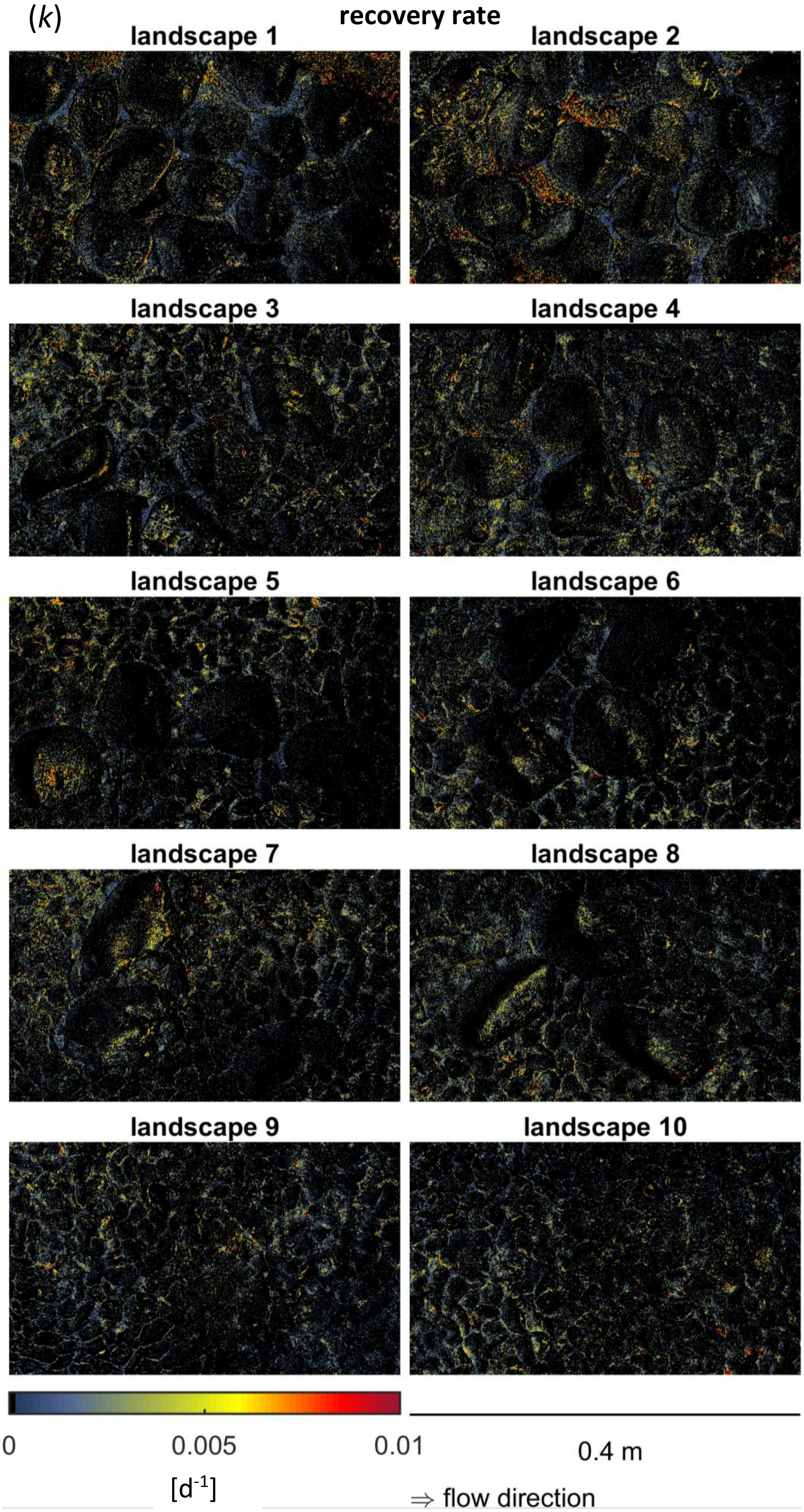
(k)
recovery rate
landscape 1
landscape 2
landscape 3
landscape 4
landscape 5
landscape 6
landscape 7
landscape 8
landscape 9
landscape 10
0
0.005
0.01
[d-1]
0.4 m
⇒ flow direction

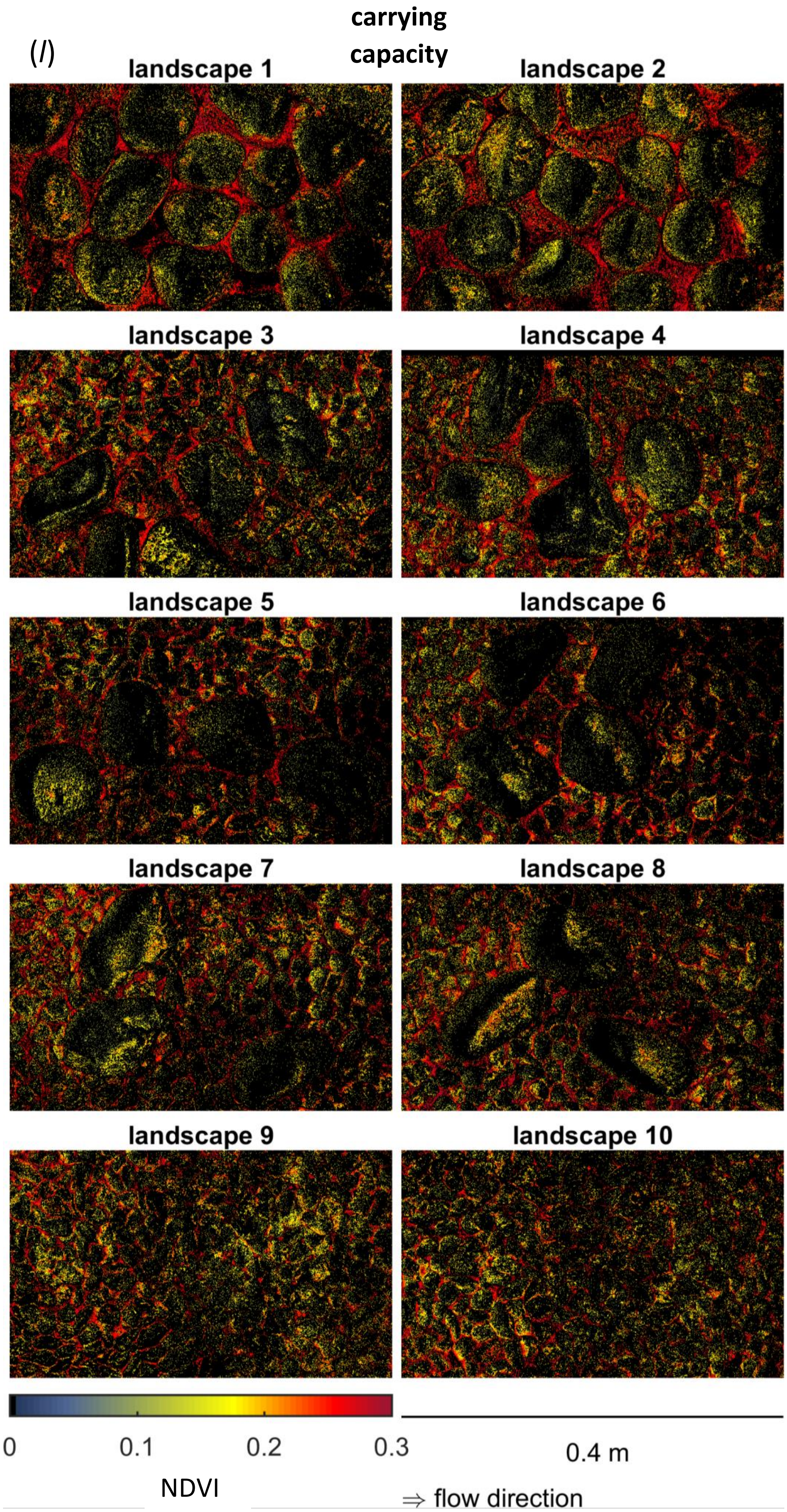
(l)
carrying capacity
landscape 1
landscape 2
landscape 3
landscape 4
landscape 5
landscape 6
landscape 7
landscape 8
landscape 9
landscape 10
0
0.1
0.2
0.3
NDVI
0.4 m
⇒ flow direction

“neighbourhood effect“

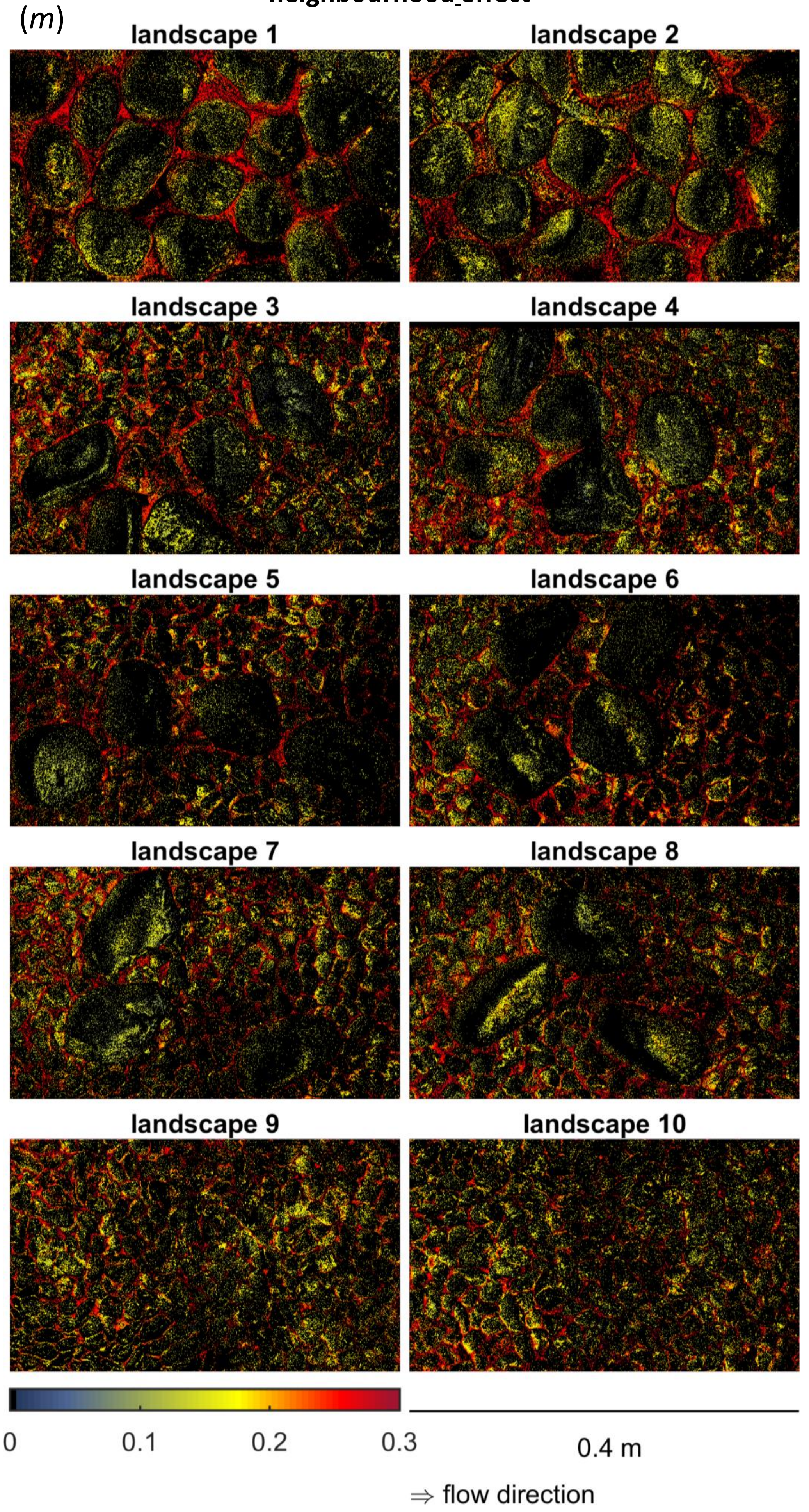

Supplementary Material S4 Response diversity (Φ) measured as the multivariate inhomogeneous intensities of lag phase, recovery rate and carrying capacity. This analysis addresses how homogenously the traits that contribute to the resilience of biofilms are distributed for each landscape without accounting for environmental variation. Thus, the same growth-related traits might be differentially expressed based on the local environmental conditions. The figures below illustrate two cases of high (blue) and low (orange) response diversity. A more homogenous distribution in trait space (i.e. blue) reflects a higher response diversity. Conceptually, we divided the trait space in increasingly smaller multivariate units (i.e. grains, illustrated by the grid) and compare the homogeneity of the distributions. Larger grains provide less resolution, but smaller grains tend to include only few datapoints. The table provides regression statistics for the 2nd order polynomial models fit between CVDEM and Φ in Figure 1 in the main text.

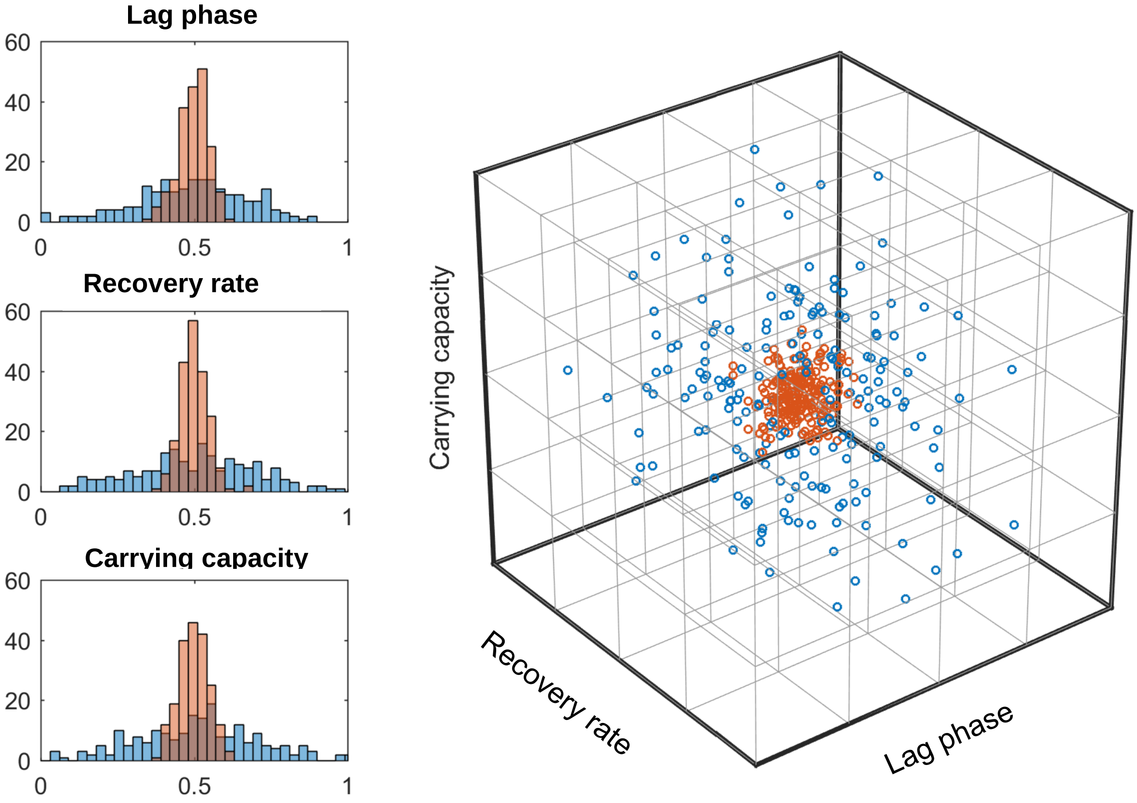

grain	convex R^2	convex p	concave R^2	concave p
5	0.45	0.05	0.11	0.28
6	0.55	0.03	-0.23	0.85
7	0.50	0.04	0.56	0.02
8	0.54	0.03	0.15	0.24
9	0.53	0.03	0.04	0.36
10	0.53	0.03	0.80	<0.01
11	0.52	0.03	0.19	0.20
12	0.52	0.03	0.37	0.08
13	0.51	0.03	0.82	<0.01
14	0.51	0.03	0.32	0.11
15	0.51	0.03	0.59	0.02
16	0.50	0.04	0.81	<0.01
17	0.50	0.04	0.45	0.05
18	0.49	0.04	0.69	<0.01
19	0.49	0.04	0.76	<0.01
20	0.49	0.04	0.56	0.02

Supplementary Material S5 Multiple linear regression models to infer relationships between growth-related parameters and environmental factors. We assessed the relative contribution of individual regressors introduced in multiple linear regression models by average-over-orders partitioning of R^2. Particularly, we used the *lmg* metric as implemented in the R package *relaimpo*. This metric decomposes R^2 into non-negative contributions that sum to the total R^2 for linear models with correlated regressor terms. This metric uses unweighted averaging over orderings to preclude that the ordering of regressors which sequentially enter the model influences their relative importance. We decomposed the relative importance for linear models explaining carrying capacity, post-disturbance biomass, lag-phase and recovery rate. Since "neighbourhood effect" and post-disturbance biomass are strongly collinear, we calculated individual models including either of these terms as regressors. Here, we provide the number of observations included for each landscape, as well as relative importance metrics and estimated coefficients for each model. We include the linear regression models as denoted in the statistical language R.

Number of observations used in the models for each landscape:

	Landscape									
	1	2	3	4	5	6	7	8	9	10
n	352029	436351	456646	467093	309444	327308	424478	388788	429124	390776

As a visual guide, we highlight relative importance for each model using the following colour gradients:

High					Low

Similarly, estimated coefficients are highlighted according to the following scheme:

Negative	Positive

CARRYING CAPACITY (post-disturbance biomass)

carrying capacity ~ post-disturbance biomass + lag phase + recovery rate + shear stress + elevation + surface slope + surface orientation + roughness + surface curvature

Relative importance metric (lmg)

	Landscape											Variance explained (%)
	1	2	3	4	5	6	7	8	9	10	Mean	
post-disturbance biomass	0.64	0.44	0.73	0.67	0.72	0.71	0.71	0.75	0.85	0.79	0.70	54.85
lag phase	0.00	0.00	0.01	0.01	0.01	0.00	0.00	0.00	0.00	0.00	0.00	0.30
recovery rate	0.03	0.03	0.03	0.02	0.03	0.03	0.03	0.02	0.03	0.04	0.03	2.40
shear stress	0.06	0.13	0.08	0.09	0.07	0.04	0.06	0.03	0.01	0.02	0.06	4.58
elevation	0.10	0.21	0.09	0.11	0.08	0.09	0.08	0.05	0.02	0.03	0.08	6.60
surface slope	0.03	0.02	0.00	0.01	0.01	0.01	0.00	0.00	0.01	0.01	0.01	0.82
surface orientation	0.01	0.01	0.00	0.00	0.00	0.01	0.02	0.01	0.00	0.00	0.01	0.39
roughness	0.02	0.05	0.02	0.04	0.03	0.04	0.03	0.03	0.01	0.01	0.03	2.16
surface curvature	0.12	0.11	0.04	0.05	0.06	0.07	0.07	0.10	0.07	0.09	0.08	6.17
**variance explained (%)**	**84.4**	**77.1**	**81.9**	**81.2**	**77.8**	**78.8**	**74.9**	**73.7**	**76.8**	**76.3**	**78.3**	**78.3**

Coefficients

	Landscape									
	1	2	3	4	5	6	7	8	9	10
post-disturbance biomass	0.87	0.71	0.91	0.87	0.88	0.85	0.85	0.85	0.86	0.83
lag phase	-0.09	-0.10	-0.10	-0.07	-0.08	-0.06	-0.05	-0.02	-0.05	-0.06
recovery rate	0.24	0.24	0.25	0.23	0.23	0.21	0.21	0.18	0.22	0.24
shear stress	0.00	0.00	-0.02	-0.02	0.05	0.07	0.10	0.13	0.02	0.01
elevation	-0.05	-0.22	-0.01	-0.03	-0.05	-0.10	-0.07	-0.08	-0.01	-0.04
surface slope	0.00	-0.01	-0.01	-0.01	-0.01	-0.01	-0.02	-0.02	0.00	0.02
surface orientation	-0.05	-0.07	-0.04	-0.05	-0.04	-0.04	-0.04	-0.04	0.01	0.03
roughness	0.05	-0.03	-0.01	0.01	0.02	0.02	0.03	0.00	0.01	0.00
surface curvature	0.10	0.08	0.04	0.09	0.07	0.06	0.10	0.11	0.08	0.10

CARRYING CAPACITY ("neighbourhood effect")

carrying capacity ~ "neighbourhood effect" + lag phase + recovery rate + shear stress + elevation + surface slope + surface orientation + roughness + surface curvature

Relative importance metric (lmg)

	Landscape											Variance explained (%)
	1	2	3	4	5	6	7	8	9	10	Mean	
neighbourhood effect	0.65	0.46	0.74	0.68	0.73	0.72	0.72	0.76	0.86	0.81	0.71	56.02
lag phase	0.00	0.00	0.00	0.00	0.00	0.00	0.00	0.00	0.00	0.00	0.00	0.25
recovery rate	0.02	0.03	0.02	0.02	0.02	0.02	0.03	0.02	0.03	0.03	0.02	1.82
shear stress	0.06	0.13	0.08	0.09	0.07	0.04	0.06	0.03	0.01	0.02	0.06	4.59
elevation	0.10	0.20	0.09	0.11	0.07	0.09	0.08	0.05	0.02	0.03	0.08	6.56
surface slope	0.03	0.02	0.00	0.01	0.01	0.01	0.00	0.00	0.01	0.01	0.01	0.82
surface orientation	0.01	0.01	0.00	0.00	0.00	0.01	0.02	0.01	0.00	0.00	0.00	0.39
roughness	0.02	0.05	0.02	0.04	0.03	0.04	0.03	0.03	0.01	0.01	0.03	2.16
surface curvature	0.12	0.11	0.04	0.05	0.06	0.07	0.07	0.10	0.07	0.09	0.08	6.10
**variance explained (%)**	**85.0**	**77.8**	**81.6**	**81.0**	**77.9**	**79.5**	**75.4**	**74.9**	**77.5**	**76.5**	**78.7**	**78.7**

Coefficients

	Landscape									
	1	2	3	4	5	6	7	8	9	10
neighbourhood effect	0.89	0.75	0.93	0.89	0.90	0.88	0.88	0.89	0.87	0.85
lag phase	-0.08	-0.09	-0.08	-0.05	-0.07	-0.05	-0.04	-0.01	-0.05	-0.05
recovery rate	0.21	0.21	0.21	0.18	0.19	0.17	0.19	0.14	0.19	0.19
shear stress	0.01	0.01	0.00	0.01	0.09	0.04	0.12	0.16	0.01	0.00
elevation	-0.03	-0.19	0.01	-0.02	-0.04	-0.04	-0.03	-0.07	0.01	-0.01
surface slope	-0.01	-0.02	-0.02	-0.02	-0.02	-0.01	-0.03	-0.03	-0.01	0.01
surface orientation	-0.05	-0.07	-0.04	-0.05	-0.04	-0.03	-0.04	-0.04	0.01	0.03
roughness	0.05	-0.02	-0.01	0.01	0.01	-0.01	0.03	-0.02	0.00	-0.01
surface curvature	0.06	0.05	0.04	0.08	0.06	0.04	0.07	0.07	0.04	0.05

POST-DISTURBANCE BIOMASS

post-disturbance biomass ~ elevation + surface slope + surface orientation + roughness + surface curvature

Relative importance metric (lmg)

	Landscape											Variance explained (%)
	1	2	3	4	5	6	7	8	9	10	Mean	
elevation	0.35	0.54	0.64	0.60	0.51	0.39	0.54	0.37	0.12	0.21	0.43	14.33
surface slope	0.15	0.08	0.03	0.05	0.06	0.06	0.03	0.03	0.16	0.08	0.07	2.44
surface orientation	0.00	0.01	0.00	0.00	0.01	0.01	0.03	0.00	0.00	0.00	0.01	0.20
roughness	0.06	0.10	0.13	0.16	0.18	0.20	0.11	0.16	0.07	0.08	0.13	4.24
surface curvature	0.45	0.28	0.20	0.19	0.24	0.34	0.29	0.43	0.65	0.63	0.37	12.47
**variance explained (%)**	**42.7**	**53.6**	**30.5**	**36.0**	**32.7**	**34.3**	**36.1**	**34.0**	**16.8**	**20.1**	**33.7**	**33.7**

Coefficients

	Landscape									
	1	2	3	4	5	6	7	8	9	10
elevation	-0.39	-0.67	-0.44	-0.47	-0.39	-0.32	-0.41	-0.28	-0.07	-0.14
surface slope	0.26	0.16	0.08	0.13	0.12	0.13	0.09	0.08	0.16	0.13
surface orientation	0.00	0.06	0.01	0.00	-0.03	-0.07	-0.11	-0.05	0.01	0.01
roughness	-0.26	-0.22	0.05	0.07	0.08	0.10	0.07	0.12	0.07	0.07
surface curvature	0.78	0.39	0.35	0.35	0.42	0.57	0.50	0.67	0.65	0.67

LAG PHASE (post-disturbance biomass)

lag phase ~ post-disturbance biomass + shear stress + elevation + surface slope + surface orientation + roughness + surface curvature

Relative importance metric (lmg)

	Landscape											Variance explained (%)
	1	2	3	4	5	6	7	8	9	10	Mean	
post-disturbance biomass	0.80	0.10	0.45	0.28	0.37	0.26	0.40	0.12	0.22	0.01	0.30	0.18
shear stress	0.01	0.07	0.09	0.29	0.04	0.21	0.09	0.31	0.09	0.20	0.14	0.09
elevation	0.02	0.14	0.19	0.15	0.10	0.10	0.05	0.13	0.04	0.13	0.11	0.06
surface slope	0.02	0.03	0.08	0.06	0.04	0.01	0.02	0.04	0.26	0.00	0.06	0.03
surface orientation	0.08	0.37	0.14	0.15	0.00	0.00	0.06	0.36	0.16	0.31	0.16	0.10
roughness	0.01	0.26	0.02	0.06	0.43	0.41	0.17	0.02	0.01	0.34	0.17	0.10
surface curvature	0.05	0.04	0.02	0.02	0.01	0.01	0.21	0.02	0.22	0.01	0.06	0.04
**variance explained (%)**	**0.7**	**0.3**	**0.8**	**0.7**	**1.5**	**0.5**	**0.3**	**0.4**	**0.4**	**0.6**	**0.6**	**0.6**

Coefficients

	Landscape									
	1	2	3	4	5	6	7	8	9	10
post-disturbance biomass	0.10	-0.02	0.06	0.05	0.09	0.05	0.05	0.03	0.02	-0.01
shear stress	0.01	0.00	0.03	-0.07	0.05	-0.07	0.04	-0.08	0.03	0.06
elevation	0.01	0.00	-0.05	0.02	-0.10	0.02	-0.02	0.05	0.00	-0.03
surface slope	-0.01	0.00	-0.03	-0.02	-0.02	-0.01	0.00	-0.01	0.03	0.00
surface orientation	0.02	0.03	0.03	0.03	0.00	0.00	-0.01	0.04	0.03	0.04
roughness	0.01	-0.03	-0.01	-0.03	-0.11	-0.07	-0.03	-0.01	0.00	-0.04
surface curvature	-0.06	0.00	-0.01	-0.03	-0.02	-0.01	-0.07	-0.02	0.06	0.01

LAG PHASE ("neighbourhood effect")

lag phase~ "neighbourhood effect" + shear stress + elevation + surface slope + surface orientation + roughness + surface curvature

Relative importance metric (lmg)

	Landscape											
	1	2	3	4	5	6	7	8	9	10	Mean	Variance explained (%)
neighbourhood effect	0.79	0.08	0.49	0.41	0.40	0.28	0.38	0.20	0.37	0.01	0.34	0.22
shear stress	0.01	0.07	0.09	0.22	0.04	0.20	0.10	0.27	0.07	0.19	0.13	0.08
elevation	0.03	0.15	0.17	0.11	0.10	0.09	0.05	0.12	0.04	0.13	0.10	0.06
surface slope	0.02	0.03	0.08	0.06	0.04	0.01	0.02	0.04	0.21	0.00	0.05	0.03
surface orientation	0.09	0.37	0.13	0.12	0.00	0.00	0.06	0.33	0.14	0.31	0.16	0.10
roughness	0.01	0.26	0.02	0.05	0.41	0.40	0.18	0.03	0.00	0.35	0.17	0.11
surface curvature	0.05	0.05	0.02	0.02	0.01	0.01	0.21	0.02	0.16	0.01	0.06	0.04
**variance explained (%)**	**0.7**	**0.3**	**0.8**	**0.9**	**1.6**	**0.5**	**0.3**	**0.4**	**0.4**	**0.6**	**0.7**	**0.7**

Coefficients

	Landscape									
	1	2	3	4	5	6	7	8	9	10
neighbourhood effect	0.10	-0.01	0.07	0.07	0.10	0.05	0.05	0.04	0.04	0.01
shear stress	0.01	0.00	0.03	-0.07	0.05	-0.07	0.04	-0.07	0.03	0.05
elevation	0.01	0.01	-0.05	0.03	-0.09	0.02	-0.01	0.05	0.00	-0.02
surface slope	-0.01	0.00	-0.03	-0.03	-0.02	-0.01	0.00	-0.02	0.03	0.00
surface orientation	0.02	0.03	0.03	0.03	0.00	0.00	-0.01	0.04	0.03	0.04
roughness	0.01	-0.03	-0.01	-0.03	-0.11	-0.07	-0.02	-0.01	0.00	-0.05
surface curvature	-0.06	-0.01	-0.01	-0.04	-0.03	-0.01	-0.07	-0.03	0.05	0.00

RECCOVERY RATE (post-disturbance biomass)

recovery rate ~ post-disturbance biomass + lag phase + shear stress + elevation + surface slope + surface orientation + roughness+ surface curvature

Relative importance metric (lmg)

	Landscape											Variance explained (%)
	1	2	3	4	5	6	7	8	9	10	Mean	
post-disturbance biomass	0.15	0.27	0.18	0.17	0.11	0.09	0.08	0.07	0.06	0.07	0.13	2.76
lag phase	0.70	0.58	0.77	0.80	0.79	0.84	0.83	0.87	0.92	0.88	0.80	17.57
shear stress	0.01	0.01	0.01	0.00	0.03	0.01	0.02	0.01	0.00	0.01	0.01	0.25
elevation	0.02	0.07	0.01	0.01	0.02	0.01	0.01	0.01	0.00	0.01	0.02	0.37
surface slope	0.00	0.00	0.01	0.00	0.01	0.02	0.01	0.01	0.00	0.01	0.01	0.16
surface orientation	0.08	0.04	0.01	0.01	0.02	0.01	0.04	0.01	0.00	0.00	0.02	0.50
roughness	0.03	0.01	0.01	0.00	0.02	0.01	0.01	0.01	0.00	0.00	0.01	0.23
surface curvature	0.01	0.01	0.01	0.01	0.01	0.00	0.00	0.01	0.01	0.02	0.01	0.16
**variance explained (%)**	**22.6**	**28.0**	**25.7**	**26.8**	**22.9**	**18.8**	**19.0**	**19.3**	**19.9**	**17.1**	**22.0**	**22.0**

Coefficients

	Landscape									
	1	2	3	4	5	6	7	8	9	10
post-disturbance biomass	-0.26	-0.45	-0.28	-0.29	-0.20	-0.18	-0.16	-0.15	-0.13	-0.14
lag phase	0.41	0.40	0.45	0.47	0.43	0.40	0.40	0.41	0.43	0.39
shear stress	0.04	0.04	0.04	-0.03	0.15	0.12	0.11	0.12	-0.03	-0.01
elevation	-0.13	-0.38	-0.14	-0.06	-0.16	-0.20	-0.13	-0.08	-0.01	-0.08
surface slope	0.02	0.01	-0.01	0.00	0.00	-0.03	-0.04	-0.04	-0.02	0.05
surface orientation	-0.14	-0.09	-0.06	-0.06	-0.06	-0.05	-0.09	-0.06	-0.01	0.01
roughness	0.07	-0.05	-0.03	0.03	-0.03	-0.04	-0.02	0.06	0.01	-0.01
surface curvature	0.09	0.14	0.10	0.12	0.09	0.05	0.07	0.11	0.09	0.14

RECOVERY RATE ("neighborhood effect")

recovery rate ~ neighborhood effect + lag phase+ shear stress + elevation + surface slope + surface orientation + roughness + surface curvature

Relative importance metric (lmg)

	Landscape											
	1	2	3	4	5	6	7	8	9	10	Mean	Variance explained (%)
neighbourhood effect	0.10	0.21	0.12	0.11	0.06	0.05	0.04	0.03	0.03	0.02	0.08	1.60
lag phase	0.74	0.65	0.83	0.86	0.83	0.89	0.86	0.91	0.96	0.94	0.85	17.47
shear stress	0.00	0.01	0.01	0.00	0.03	0.01	0.02	0.02	0.00	0.01	0.01	0.24
elevation	0.02	0.06	0.01	0.00	0.02	0.01	0.01	0.01	0.00	0.01	0.02	0.32
surface slope	0.00	0.00	0.01	0.00	0.01	0.02	0.01	0.01	0.00	0.01	0.01	0.16
surface orientation	0.09	0.04	0.01	0.01	0.02	0.02	0.04	0.01	0.00	0.00	0.02	0.50
roughness	0.04	0.01	0.01	0.00	0.02	0.01	0.01	0.01	0.00	0.00	0.01	0.23
surface curvature	0.01	0.01	0.00	0.00	0.00	0.00	0.00	0.00	0.00	0.01	0.01	0.11
**variance explained (%)**	**21.2**	**25.3**	**23.9**	**24.7**	**21.6**	**17.8**	**18.2**	**18.4**	**19.2**	**16.1**	**20.6**	**20.6**

Coefficients

	Landscape									
	1	2	3	4	5	6	7	8	9	10
neighbourhood effect	-0.21	-0.40	-0.24	-0.23	-0.15	-0.13	-0.12	-0.10	-0.09	-0.09
lag phase	0.41	0.41	0.45	0.47	0.43	0.40	0.40	0.41	0.43	0.39
shear stress	0.04	0.04	0.05	-0.02	0.16	0.11	0.12	0.12	-0.03	-0.02
elevation	-0.12	-0.36	-0.13	-0.04	-0.15	-0.17	-0.12	-0.07	-0.01	-0.07
surface slope	0.01	0.00	-0.02	-0.01	-0.01	-0.04	-0.04	-0.04	-0.02	0.04
surface orientation	-0.14	-0.09	-0.06	-0.06	-0.06	-0.05	-0.09	-0.05	-0.01	0.01
roughness	0.08	-0.04	-0.03	0.03	-0.04	-0.04	-0.02	0.05	0.01	-0.02
surface curvature	0.06	0.13	0.08	0.10	0.07	0.02	0.05	0.08	0.07	0.11

Supplementary Material S6 We built a piecewise structural equation model based on the structure as suggested from multiple linear regression analyses (Supplementary Material S5). Relationships explaining at least 1% of variance in the multiple linear regressions were entered into a first SEM. A piecewise SEM includes additional, i.e. missing paths, which are supported by statistical significance. Based on biological knowledge, we excluded effects of “neighbourhood effects” and lag phase on post-disturbance biomass. Here, we report on the initial SEM which was built based on the multiple linear regression analyses and the updated SEM after inclusion of missing paths. For each SEM, the underlying generalized mixed effects models, evaluating the relationships between post-disturbance biomass, recovery rate and carrying capacity with the measured factors are denoted in the statistical language R. Model family, link type, number of datapoints and R^2 are provided. Missing paths as suggested after the initial SEM and predictors and responses and predictors for initial and final SEM are shown. Goodness of fit tests included Akaike Information Criterion (AIC) and Fishers C, which evaluates independences claims.

INITIAL SEM

	class	family	link	n	R^2
post-disturbance biomass ~ elevation + surface slope + roughness + surface curvature	lm	gaussian	identity	3982037	0.29
recovery rate ~ post-disturbance biomass + “neighbourhood effect” + lag phase	lm	gaussian	identity	3982037	0.22
carrying capacity ~ post-disturbance biomass + “neighbourhood effect” + recovery rate + shear stress + elevation + roughness + surface curvature	lm	gaussian	identity	3982037	0.78

**missing path**	**estimate**	**std. error**	**df**	**critical value**	**p**
recovery rate ~ elevation + ...	-0.0028	0.0001	3982032	-36	<0.01
recovery rate ~ surface slope + ...	-2.2218	0.1226	3982032	-18	<0.01
carrying capacity ~ surface slope + ...	-118.958	1.4111	3982028	-84	<0.01
recovery rate ~ roughness + ...	0.0000	0.0000	3982032	74	<0.01
recovery rate ~ surface curvature + ...	0.0002	0.0000	3982032	104	<0.01
*post-disturbance biomass ~ neighbourhood effect + ...	1.0632	0.0001	3982031	7101	<0.01
*post-disturbance biomass ~ lag phase + ...	0.0005	0.0000	3982031	85	<0.01
carrying capacity ~ lag phase + ...	-0.0009	0.0000	3982028	-260	<0.01
post-disturbance biomass ~ shear stress + ...	5.5831	0.1227	3982031	46	<0.01
recovery rate ~ shear stress + ...	0.0612	0.0027	3982032	22	<0.01

* excluded

**response**	**predictor**	**estimate**	**std. error**	**p**
post-disturbance biomass	elevation	-0.3148	0.000523	<0.01
post-disturbance biomass	surface slope	0.1653	0.000442	<0.01
post-disturbance biomass	surface heterogeneity	0.0275	0.000509	<0.01
post-disturbance biomass	surface curvature	0.5830	0.000924	<0.01
recovery rate	post-disturbance biomass	-0.7787	0.001939	<0.01
recovery rate	neighbourhood	0.6292	0.001939	<0.01
recovery rate	lag phase	0.4140	0.000444	<0.01
carrying capacity	post-disturbance biomass	0.3917	0.001039	<0.01
carrying capacity	neighbourhood	0.4767	0.001054	<0.01
carrying capacity	recovery rate	0.1897	0.000239	<0.01
carrying capacity	elevation	-0.0301	0.00061	<0.01
carrying capacity	surface heterogeneity	0.0184	0.000271	<0.01

carrying capacity	surface curvature	0.0716	0.00054	<0.01
carrying capacity	shear stress	0.0206	0.000563	<0.01

FINAL SEM

	class	family	link	n	R^2
post-disturbance biomass ~ shear stress + elevation + surface slope + roughness + surface curvature	lm	gaussian	identity	3982037	0.29
recovery rate ~ post-disturbance biomass + neighbourhood effect + lag phase + shear stress + elevation + surface slope + roughness + surface curvature	lm	gaussian	identity	3982037	0.22
carrying capacity ~ post-disturbance biomass + neighbourhood effect + lag phase + recovery rate + shear stress + elevation + surface slope + roughness + surface curvature	lm	gaussian	identity	3982037	0.79

response	predictor	estimate	std. error	p
post-disturbance biomass	shear stress	0.046	0.001	<0.01
post-disturbance biomass	elevation	-0.359	0.001	<0.01
post-disturbance biomass	surface slope	0.166	0.000	<0.01
post-disturbance biomass	roughness	0.025	0.001	<0.01
post-disturbance biomass	surface curvature	0.578	0.001	<0.01
recovery rate	post-disturbance biomass	-0.765	0.002	<0.01
recovery rate	neighbourhood	0.590	0.002	<0.01
recovery rate	lag phase	0.415	0.000	<0.01
recovery rate	shear stress	0.113	0.001	<0.01
recovery rate	elevation	-0.095	0.001	<0.01
recovery rate	surface heterogeneity	0.034	0.001	<0.01
recovery rate	surface curvature	0.092	0.001	<0.01
recovery rate	surface slope	-0.014	0.000	<0.01
carrying capacity	post-disturbance biomass	0.407	0.001	<0.01
carrying capacity	neighbourhood	0.474	0.001	<0.01
carrying capacity	lag phase	-0.066	0.000	<0.01
carrying capacity	recovery rate	0.218	0.000	<0.01
carrying capacity	surface slope	-0.021	0.000	<0.01
carrying capacity	surface heterogeneity	0.023	0.000	<0.01
carrying capacity	surface curvature	0.068	0.001	<0.01
carrying capacity	elevation	-0.020	0.001	<0.01
carrying capacity	shear stress	0.017	0.001	<0.01

	Fisher's C	df	p	AIC	K	n
Initial SEM	2676.4	20	<0.01	2716.4	20	3982037
Final SEM	144.59	4	<0.01	200.59	28	3982037

Supplementary Material S7 One-sample Wilcoxon rank sum test statistics to compare median Φ_{convex}:$\Phi_{concave}$ against a value of 1. For each landscape estimated medians, W (Wilcoxon test statistics) and p values are shown. Note that the ratios of response diversity in convex and concave landscape components (Φ_{convex}:$\Phi_{concave}$) were significantly larger than 1 for both landscapes composed of only large sediments (landscape 9 and 10). The ratios of response diversity in landscapes composed only of small sediments (landscape 1 and 2) were marginally larger and smaller than 1, whereas the ratios were significantly smaller than 1 in all landscapes composed of mixtures of small and large sediments.

	Landscape									
	1	2	3	4	5	6	7	8	9	10
median	1.04	0.92	0.88	0.86	0.89	0.89	0.92	0.98	1.09	1.09
W	111	120	136	136	136	136	136	123	136	136
p-value	0.03	0.007	< 0.001	<0.001	0.001	<0.001	<0.001	0.004	<0.001	<0.001
